# Expanding the Chemical Space of Benzimidazole Dicationic Ionic Liquids

**DOI:** 10.3390/molecules26144211

**Published:** 2021-07-11

**Authors:** Andrea Mezzetta, Luca Guglielmero, Angelica Mero, Giorgio Tofani, Felicia D’Andrea, Christian Silvio Pomelli, Lorenzo Guazzelli

**Affiliations:** 1 Department of Pharmacy, University of Pisa, Via Bonanno 33, 56126 Pisa, Italy; luca.guglielmero@phd.unipi.it (L.G.); angelica.mero@phd.unipi.it (A.M.); giorgio.tofani@df.unipi.it (G.T.); felicia.dandrea@farm.unipi.it (F.D.); christian.pomelli@unipi.it (C.S.P.); lorenzo.guazzelli@unipi.it (L.G.); 2 DESTEC, University of Pisa, Largo Lucio Lazzarino, 56122 Pisa, Italy; 3 Department of Physics, University of Pisa, Largo Bruno Pontecorvo 3, 56127 Pisa, Italy

**Keywords:** ionic liquids, green solvents, benzimidazole, dicationic ionic liquids, thermal stability, thermal behavior, cyclic voltammetry

## Abstract

Benzimidazole dicationic ionic liquids (BDILs) have not yet been widely explored in spite of their potential. Therefore, two structurally related families of BDILs, paired with either bromide or bistriflimide anions and bearing alkyl spacers ranging from C3 to C6, have been prepared. Their thermal properties have been studied by thermogravimetric analysis (TGA) and differential scanning calorimetry (DSC), while their electrical properties have been assessed by cyclic voltammetry (CV). TG analysis confirmed the higher stability of the bistriflimide BDILs over the bromide BDILs, with minor variation within the two families. Conversely, DSC and CV allowed for ascertaining the role played by the spacer length. In particular, the thermal behavior changed dramatically among the members of the bistriflimide family, and all three possible thermal behavior types of ILs were observed. Furthermore, cyclic voltammetry showed different electrochemical window (C_3_(C_1_BenzIm)_2_/2Tf_2_N < C_4_(C_1_BenzIm)_2_/2Tf_2_N, C_5_(C_1_BenzIm)_2_/2Tf_2_N < C_6_(C_1_BenzIm)_2_/2Tf_2_N) as well as a reduction peak potential, shape, and intensity as a function of the spacer length. The results obtained highlight the benefit of accessing a more structurally diverse pool of compounds offered by dicationic ILs when compared to the parent monocationic ILs. In particular, gains are to be found in the ease of fine-tuning their properties, which translates in facilitating further investigations toward BDILs as designer solvents and catalysts.

## 1. Introduction

In the last 20 years, ionic liquids (ILs) have been undoubtedly a “hot” topic in the field of sustainable chemistry [1]. The number of ILs studied has grown constantly, and their use has spread in all branches of science, from chemistry [2] and engineering [3] to biology and medicine [4]. The reason for this sustained interest in ILs lies in their structural variability, which makes it possible to design suitable matching ions for a given application [5]. Although ILs exhibit unique properties and potential such as unmatched ability to dissolve several biopolymers [6,7], high thermal stability [8,9], high ability to solubilize gas [10], and high refractive index [11], the reputation of ILs as “green” solvents is certainly ascribable to their negligible vapor pressure, which makes them safe when compared to traditional organic solvents [12,13]. In fact, ILs are practically non-flammable [14], and the possibility of accidental spills is negligible. However, the environmental footprint and toxicity of ILs have been analyzed over time under different perspectives leading to a re-evaluation of the effective, sustainable nature of at least some of these media. To improve ILs’ sustainability-related issues, synthetic procedures for their preparation have been optimized, focusing on the replacement or elimination of polluting organic solvents and on the usage of reagents deriving from renewable sources [15]. In parallel to this approach, toxicity and biodegradation assessments pointed to structural elements of the cationic and anionic parts capable of ameliorating the overall impact profile of ILs [16,17]. This new type of ILs, often referred to as the fourth generation of ILs, allowed for expanding the portfolio of possible applications in the fields of food science [18,19], medicine [20,21] and pharmaceutics [22,23].

On the other hand, the development of new structures, the exploration of the intertwining interactions between cation and anion, and the understanding of the relationship between the structure and the physico-chemical properties of the solvent are with no doubt other heavily studied areas in the field of ILs. In this context, dicationic ionic liquids (DILs) allowed for a broadening of the structural variability of ILs. DILs are composed of two cations connected by a suitable linker and paired with two counteranions [24]. Although linear spacers are the most commonly found in literature, linkers of different nature have also been reported, such as branched alkyl [25], polyethers [26], aromatic chains [27,28,29], and fragments derived from natural sources [30]. Compared to the parent monocationic ILs, DILs exhibit superior tunability and present different structural organizations [31]. These peculiar features often result in lower polarity, higher thermal stability [28,32], higher glass transition temperatures and melting points [33,34,35], larger surface tensions, higher shear viscosities [36], and lower ecotoxicity when compared to monocationic ILs [37]. These properties allowed DILs to be used effectively in a wider portfolio of applications, including catalysis [38,39], high-temperature batteries [40], high-temperature lubricants [41], stationary phases for chromatography [42], and dye-sensitized solar cells [43].

In addition to the structural modifications of specific ions, the search for new cations is key for the fine-tuning of ILs properties. In this framework, benzimidazolium-based ILs (BILs) have been certainly understudied with respect to the vast majority of cations described in literature, such as imidazolium, pyridinium, pyrrolidinium, and morpholinium [44]. Starting from the first report by Katritzky et al. [45], BILs were investigated in fields that comprise CO_2_ capture and conversion [46,47], electronics [48,49,50], dye solar cells [51], anticancer and antibacterial agents [52,53], bioremediation technologies [54,55], fuel desulfurization [56], extraction [57], lignin conversion [58], and fuel cell [59,60]. On the other hand, an area in which BILs were heavily studied is organic synthesis, where they were successfully used as catalysts in several reaction classes such as the Biginelli reaction [61], the Micheal reaction [62,63], condensations [64,65,66,67], alkylations [68], amine formylation [69], coupling [70], and esterifications [71]. Furthermore, the use of BILs as catalysts has also been reported in the preparation of natural-derived building blocks: in the transesterification of castor oil with methanol [72], in the production of ethyl levulinate [73] and in the preparation of 5-hydroxymethyl furfural starting from chitosan [74].

The benzimidazole nucleus has also been used for the preparation of benzimidazole dicationic ionic liquids (BDILs). Similar to what was mentioned above, the use of dicationic structures allows for increasing the modulation ability of the BILs. Although the number of reported papers is limited, BDILs have been employed in a large number of fields such as in analytics [75], metal extractions [76], as chelating agents [77], pharmaceutics [78] and surfactants [79,80]. One of the first applications of BDILs was against microbial agents, both neat [81,82] and coated on multiwalled carbon nanotube [52]. In the pharmaceutical field, Clark et al. [83] reported the effective use of magnetic BDILs in DNA extraction for analytical purposes. In addition, for BDILs, the majority of the attention has been paid by the organic chemistry community, with an emphasis on their role as reaction catalysts. BDILs have been used in many transformations spanning from Heck [84], Suzuki [85], Benzoin [86], and Kumada couplings [87] to desulfurization reactions [88], and esterifications [89]. Furthermore, by treating a DBIL with a strong base, a cyclic “dimer” can be formed, which is highly reactive toward air and can be used as an organic super-electron donor [90]. In the case of the imidazole dicationic ILs, the reaction is less favorable, and it is necessary to start from a cyclic dicationic species with a more demanding synthesis [91]. These compounds have been used as single and double electron transfer systems in organic reactions [92,93] and as initiators for radical polymerizations [94].

Despite the promising potential shown by DBILs, they are still underexplored with respect to their imidazole analogs. This holds true both in terms of their practical applications and of their physico-chemical properties, and their changes as a function of the chemical structure. For the above reasons, in the present work, we report the study of the thermal and electrical properties of two structurally related series of BDILs with bromide and bis(trifluoromethane)sulfonimide as anions.

## 2. Results and Discussion

### 2.1. Synthesis of Benzimidazole Dicationic Ionic Liquids

**Scheme 1:**
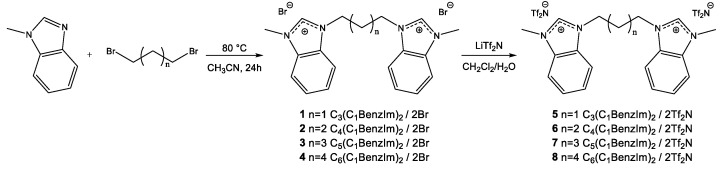
Synthetic procedure for the synthesis of benzimidazole dicationic ionic liquids **1–8**.

A series of structurally related dibromides benzimidazole dicationic ionic liquids (**1–4**), varying in the length of the alkyl spacer between the cationic headgroups from C3 to C6, was synthesized via the Menschutkin reaction between *N*-methylbenzimidazole and different dibromoalkane (Scheme 1). Starting from the dibromide precursors, C_n_(BenzIm)_2_ / 2Tf_2_N **5–8** were then prepared through metathesis reaction using lithium bis(trifluoromethane)sulfonimide (Scheme 1).

The chemical structures of benzimidazolium salts (**1–8**) were assessed by NMR experiments (^1^H and ^13^C), and all spectral data were in accordance with the proposed structures. For bis(trifluoromethane)sulfonimide BDILs the complete anion exchange and removal of the halide anion were evaluated by means of the silver nitrate test. In the ^13^C NMR spectrum of compounds **5–8**, the presence of the quadruplet signal (around 121 ppm, *J*_C,F_ about 320 Hz), ascribable to the CF_3_ group, confirmed the presence of the bis(trifluoromethane)sulfonimide anion. It is worth noting that in the ^1^H spectra of compounds **1–8**, the signal of the H-2 (NC*H*N, 9.69–9.29) attributable to benzimidazolium core decreases as a function of time (see Supplementary Information, Figures S7 and S11). This phenomenon is due to the fact that the H-2 of the imidazolium ring exchanges with the deuterium of the solvent if a protic solvent is used as CD_3_OD in the present case [95,96]. The ^13^C NMR spectra confirmed the presence of the deuterium at the carbon C-2, giving a triplet around 143 ppm (*J* about 33.5–32.5 Hz) (see Supplementary Information, S14).

### 2.2. Thermal Gravimetric Analysis (TGA)

**Figure 1:**
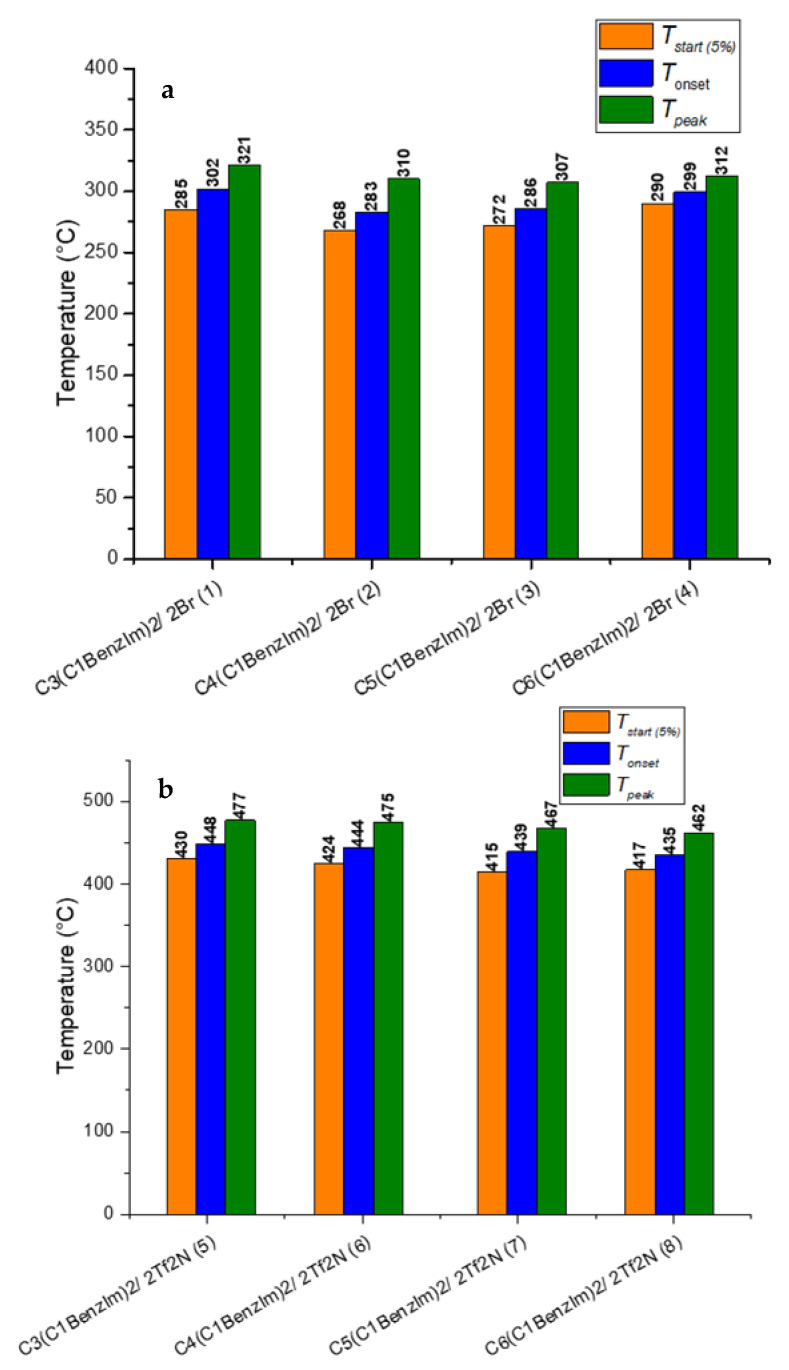
*T*_start_*, T*_onset,_ and *T*_peak_ for the bromide (**a**) and the bistriflimide (**b**) BDILs at a heating rate of 10 °C/min.

**Figure 2:**
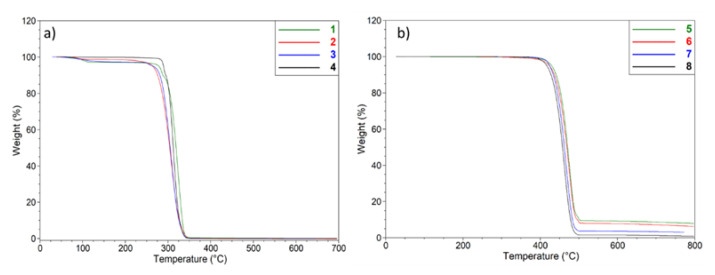
Thermal gravimetric analysis of bromide (**a**) and Tf_2_N BDILs (**b**).

The thermal stability of all synthesized BDILs was studied by thermal gravimetric analysis. All samples were accurately dried before the analysis and subjected to a further drying cycle consisting of an isothermal run at 60 °C for 30 min. The BDILs were exposed to a linear increase in temperature (10 °C/min) up to 700 °C, and the mass loss was recorded. Three typical thermal stability parameters *T*_start_, *T*_onset,_ and *T*_peak_ were obtained from the thermograms for each investigated BDIL. The data registered are reported in Figure 1 and Table S1. A comparison between the curves obtained for bromide based-BDILs (**1–4**) and bistriflimide based-BDILs (**5–8**) is shown in Figure 2. The single TG and DTG curves of each compound are reported in the Supplementary Information (Figures S23–S30).

All Tf_2_N BDILs were characterized mainly by one degradation process with decomposition temperatures that slightly decreased as the length of the linker increased. In this context, Kermanioryani et al. studied the physico-chemical properties of novel imidazolium-based ILs, among which the monocationic butyl-benzimidazolium bistriflimide [BzBIm]Tf_2_N. The authors reported a *T*_onset_ of 421.52 °C for this latter IL, a lower value if compared to the corresponding dicationic BDILs with a C_4_ linker (**6**) synthesized in this work. This suggests higher thermal stability for dicationic benzimidazolium ILs with respect to the monocationic ones [97]. Furthermore, a comparison with Tf_2_N imidazolium dicationic ILs, previously synthesized by our group, allows for interesting considerations [35]. All samples were thermally characterized using the same instrument and analysis conditions employed in this work. It was observed that all the Tf_2_N imidazolium dicationic ILs with linkers of different lengths (C3 or C6) bearing different lateral substituents (C4 or C8) showed higher thermal stability (*T*_onset_ ranging between 467 °C and 478 °C) than the Tf_2_N BDILs proposed here. A similar trend was reported by Al-Mohammed et al*.,* who synthesized and characterized bis-imidazolium and benzimidazolium gemini-type ILs containing the sulphamide moiety in the linker and six different lateral chains [82]. Indeed, they observed that imidazolium DILs, with chloride and bromide anions, generally displayed higher thermal stability than ILs with benzimidazolium cations.

On the other hand, all bromide BDILs thermograms presented the main degradation step with a small shoulder at the beginning of the process. Moreover, the bromide salts **2** and **3** displayed a lower *T*_start_ than **1** and **4** due to the mass loss below 100 °C, which is ascribable to the evaporation of the absorbed and bounded water. Indeed, despite the accurate drying process before the analysis, bromide BDILs are difficult to dry due to their hygroscopic character. When the bromide BDILs were compared to the bromide methylimidazolium dicationic ILs, also synthesized by our group with the same alkyl linker lengths (from C3 to C6), they did not show substantial differences regarding the thermal stability. Indeed, the methylimidazolium DILs displayed *T*_onset_ ranging from 282 to 298 °C [98].

Following the thermal stability classification based on the *T*_onset_ values of 66 different ILs described by Cao and Mu, who identified five possible stability levels [99], the bromide BDILs **1–4** belong to the “less stable” class (250 °C < *T*_onset_ < 300 °C) while the Tf_2_N BDILs **5–8** belong to the “most stable” class (400 °C < *T*_onset_ < 450 °C).

### 2.3. Differential Scanning Calorimetry (DSC)

**Figure 3:**
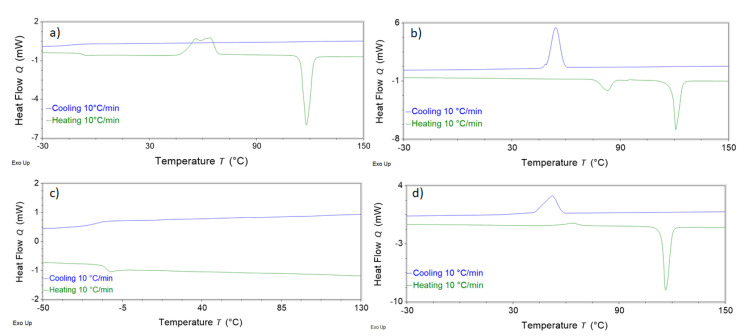
Differential scanning calorimetry (DSC) of compounds **5** (**a**), **6** (**b**), **7** (**c**), and **8** (**d**).

The thermal behavior of all synthesized BDILs was explored by differential scanning calorimetry analyses performed at different scanning rates (2–20 °C/min) under nitrogen atmosphere and in the temperature range going from −90 to 200 °C. In particular, for bromide BDILs a first heating run was registered at 10 °C/min, and the melting of the hydrated form was clearly observed with the exclusion of compound **4** (Table S2). Next, all samples were subjected to the second cycle of cooling-heating at 10 °C/min. All the thermograms of bromides BDILs **1–4** are reported in the Supplementary file (S31–34, Table S3). Compound **4** did not show any transition, suggesting a degradation step before melting. Compounds **1** and **3** displayed only the melting of the hydrated form during the first heating run, while they did not show any transition in the second cycle. Finally, compound **2** presented both the melting of the hydrated form and of the anhydrous form in the first heating run, while it showed only a glass transition at 73 °C in the second cycle. It is worth noting how the corresponding dicationic methylimidazolium bromides ILs showed a more complex thermal behavior, generally characterized by a glass transition or a solid-solid transition, a cold crystallization, and a melting process during the heating run [98]. For what concern the Tf_2_N based-BDILs, after the second cycle at 10 °C/min, the third cycle of cooling-heating at 5 °C/min was registered. Compound **7** was also subjected to three additional cooling-heating cycles at 15, 20, and 2 °C/min to investigate in-depth its thermal behavior. The second cycle of all the Tf_2_N BDILs is reported in Figure 3, while the other thermograms, including the comparison between the different scanning rates, are reported in the Supplementary Information (S35–S38).

Gómez et al. [100] reported three thermal behaviors usually observed for ILs: (i) salts, which display only a glass transition (type I); (ii) ILs behaving as common salt showing the melting transition upon heating and the corresponding freezing transition upon cooling (type II); and (iii) ILs that do not show a tendency to crystallize upon cooling, which, however, display a cold crystallization on heating (type III). For the investigated Tf_2_N BDILs whose only difference is the length of the linker between the benzimidazolium heads, all three thermal behaviors were found. Indeed, as can be observed from Figure 3, BDILs **6** and **8** behave as type II ILs. More specifically, **6** presented two melting phenomena (83 °C and 121 °C) during the heating run and a single crystallization upon the cooling run at all tested heating rates. Similarly, **8** was characterized by melting and related freezing transitions. A small cold crystallization was also visible on both the heating runs registered at 10 °C/min and at 5 °C/min. Compound **5** displayed, instead, a typical type III thermal behavior. Indeed, it did not exhibit the formation of crystals during the cooling run corresponding to the melting transition, but a cold crystallization was clearly observed following a glass transition in the heating run. This behavior was noticed at all different heating rates. Finally, BDIL **7**, after exhibiting a melting transition at 88 °C during the first heating at 10 °C/min, showed only a glass transition at all the different scanning rates examined (2, 5, 10, 15 and 20 °C/min) without any other phase transition, thus indicating a type I behavior. More precisely, the first run showed melting of the sample obtained as a solid from its preparation by adding an antisolvent to the reaction mixture. Instead, when the melted sample was slowly cooled to −90 °C, it did not exhibit the formation of a crystalline form in the cooling run while a glass transition appeared in the subsequent heating run. It is interesting to note how decreasing the scanning rate from 20 to 2 °C/min caused the *T*_g_ values to slightly increase (from −17.6 to −13.9 °C, respectively). As a result of this behavior, it was possible to determine BDIL **7′**s apparent activation energy (*E*_a_) for the relaxation times controlling the structural enthalpy relaxation and consequently its fragility index (*m*), an important parameter correlated to the conductivity of an IL [101]. It is known that studying the variation of *T*_g_ as a function of the heating rate (β) allows for an estimation of *E*_a_ and thus the *m* parameter. Therefore, in the Supplementary file, the logarithm of the heating rate (lnβ) has been plotted as a function of 1/T. A linear trend was obtained, and *E*_a_ and *m* were determined (Table S4) through the following equations:

(1) dlnβd1Tg= −EaR

(2) $$m=\;\frac{1}{2.303}\;\left[\frac{E_{a}\left(T_{g}\right)}{RT_{g}}\right]$$

Compared to the corresponding dicationic C_5_(C_1_Im)_2_/2Tf_2_N studied by Sippel et al. [102] and to other Tf_2_N dicationic imidazolium ILs with different alkyl chain length on the imidazole ring reported previously by our group [35], BDIL **7** displays higher *T*_g_ and lower *m* values. Consequently, it could be assumed that this BDIL will show lower conductivity than the corresponding imidazolium system. Indeed, the highest conductivities were obtained for ILs characterized by both a high fragility index and a low *T*_g_.

Lastly, the already reported influence of the temperature scanning rate on the thermal behavior of an IL [103] was observed here: higher rates caused the glass transition and cold crystallization temperatures to increase while the freezing temperature was reduced. Instead, the melting transition was not influenced by the different scanning rates.

**Figure 4:**
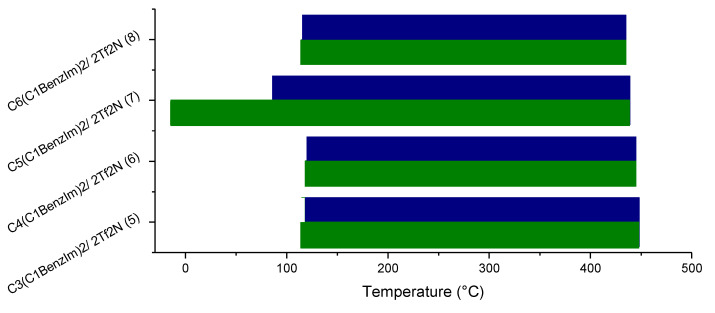
Operative range of Tf_2_N BDILs: the blue bars and green bars were obtained by using a minimum value of the temperature of the transition of the first heating run and the second heating run of DSC analyses, respectively. As the maximum value of temperature, *T*_onset_ of the main degradation obtained by TGA analyses was taken in both cases.

The data obtained from the thermal analyses allowed the assessment of the operative range of these new BDILs, as shown in Figure 4. The *T*_onset_ of melting transition, gathered from DSC analyses, was taken as the minimum value, while the *T*_onset_ of the main degradation registered in the TGA analyses was taken as the maximum applicable temperatures. It is worth stressing that no substantial differences can be noted when the linker between the two benzimidazolium heads is varied, except for compound **7**. Indeed, this specific system showed a wider operative range both considering the melting temperature registered in the first heating run (blue bar) and especially the glass transition observed in the second heating run (green bar) of DSC analysis.

### 2.4. Electrochemical Window Determination

**Figure 5:**
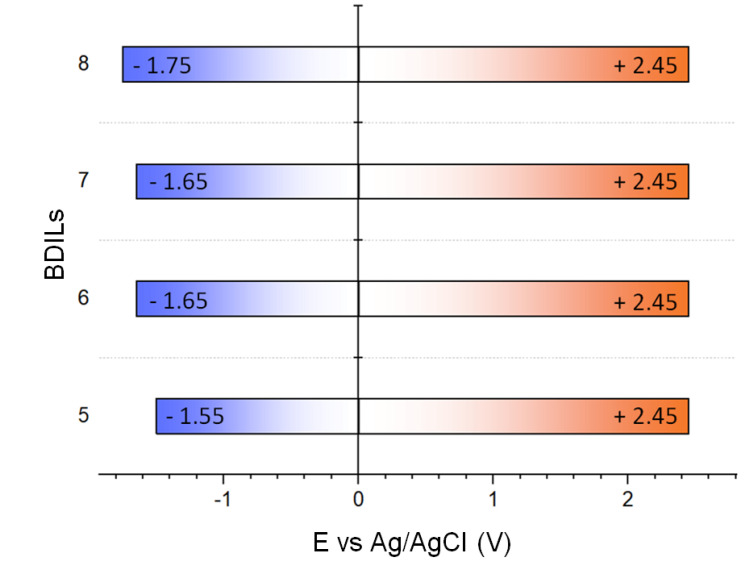
ECW comparison of BDILs **5**–**8**.

The electrochemical window (ECW) of BDILs **5**–**8** has been estimated through cyclic voltammetry of 0.1 M solution in acetonitrile, using a platinum working electrode. The results are reported in Figure 5.

**Figure 6:**
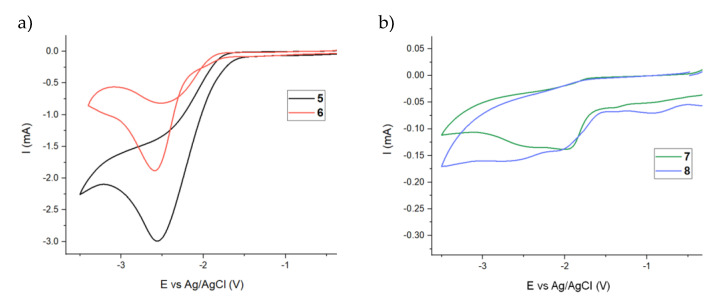
(**a**) Cyclic voltammetries of BDILs **5** and **6** 0.01 M in MeCN at 100 mV s^−1^; (**b**) cyclic voltammetries of BDILs **7** and **8** 0.01 M in MeCN at 100 mV s^−1^.

Although BDILs **5**–**8** showed very similar electrochemical stability properties, it was possible to appreciate a slight widening of the ECW with the elongation of the linker chain. Compound **5** displayed an ECW of about 4 V, **6** and **7** of 4.1 V and **8** was found to exhibit the largest ECW with 4.2 V. Having all the considered BDILs the same resistance toward oxidation, the only differences could be appreciated on the cathodic side of the ECW, related with the cation stability toward reduction [104,105,106]. A few studies have already been reported where the cathodic behavior of imidazolium ILs are presented: [104,105] they indicate the possibility of the formation of N-heterocyclic carbene (NHC) species at potential lower than −2 V vs. Ag/AgCl. More specifically, the reduction in dicationic systems has been described as influenced by the length of the linker chain connecting the imidazolium groups, suggesting a cooperative effect between the aromatic rings [105]. Consistent with these data, the cyclic voltammetry experiments of the benzimidazolium DILs reported in this work also show a strong dependence on the linker chain. Interestingly, not only the reduction peak potential was affected, but also its shape and intensity (Figure 6). BDILs **7** and **8** showed a composite reduction peak and, symmetrically, an enlarged oxidation signal centered around the 0.25–0.50 V region. The elongation of the linker chain appears to hinder the simultaneous reduction in the two benzimidazolium groups observed for the shorter chain BDILs **5** and **6**, resulting in the manifestation of a double reduction and of the related oxidation signals.

Concerning the NHC reduction product, it is possible to consider, on the basis of literature data, the formation of tetraazafulvene-like cyclic structures through the intramolecular coupling of the carbenes. Toward this end, cyclic voltammograms between −3.5 V and +2 V (potential within which NHC or its related dimer should be oxidized) have been analyzed. It is reported by Broggi et al. [94] that the NHC dimer reduction potential is at about −0.76 V; however, no clear signals were found in the region. These results confirm the absence of bridged cation formation but cannot help drawing any conclusion on the formation of the neutral dimer under our experimental conditions.

## 3. Materials and Methods

### 3.1. Material and Instruments

1-Methylbenzimidazole (>98%) was purchased from TCI Chemicals. 1,3-Dibromopropane (99%), 1,4-dibromobutane (99%), 1,5-dibromopentane (97%), 1,6-Dibromohexane (96%), acetonitrile (99.5%), diethyl ether (99.7%) and dichloromethane (99.8%), were obtained from Merck Life Science S.r.l. (Darmstadt, Germania) Lithium bis(trifuoromethylsulfonyl)imide was purchased from Merck Life Science S.r.l. (Darmstadt, Germania) All reagents were used as received without further purification.

NMR spectra were recorded with a Bruker Avance II (Bruker Italia S.r.l., Milano, Italy) operating at 400 (^1^H) and 100 MHz (^13^C) and using deuterated methanol as solvent. Chemical shifts (δ) are referenced to the residual solvent signal of CD_3_OD (^1^H 3.31 ppm, ^13^C 49.0 ppm) while coupling constants (*J*) are expressed in Hertz (Hz). The following abbreviations are used: s = singlet, m = multiplet, t = triplet, q = quartet.

The thermal stability of the synthesized ILs was investigated by thermal gravimetric analysis (TGA), conducted in a TA Instruments Q500 TGA (New Castle, DE, USA) equipped with an EGA(Evolved Gas Analysis) furnace. The thermal behavior of the ionic liquids was analyzed by a differential scanning calorimeter TA Instruments DSC, Q250 (TA Instruments, New Castle, DE, USA).

Cyclic voltammetry (CV) analysis was performed with a conventional three-electrode cell equipped with a reference electrode (Ag/AgCl 3 M KCl, Amel 373/SSG/6), a counter electrode (platinum wire, Amel 805/SPG/12), and a working electrode consisting of a glassy carbon disk (GC, 0.07 cm^2^ area). A VMP3 Biologic Science Instrument device was used as a potentiostat (EC-lab V10.18 software was used to control the potentiostat).

### 3.2. Synthesis of DILs

#### 3.2.1. General Procedure for Synthesis of Bromide Benzimidazole Dicationic Ionic Liquids (Bdils)

The bromide benzimidazole dicationic ionic liquids (BDILs) were obtained following a general procedure previously reported [38]. To a solution of opportune 1,n-dibromoalcane (1 mol) in dry acetonitrile (25 mL), a solution of commercial *N*-methylbenzimidazole (2.6 mol in 20 mL of dry acetonitrile) was added, and the reaction mixture was heated to 80 °C and stirred under nitrogen atmosphere. After 24 h, the solution was cooled to room temperature, diethyl ether (Et_2_O, 30 mL) was added, and immediately, the precipitation of a white solid was observed. Solid precipitates were filtrated under vacuum, washed with diethyl ether (3 × 50 mL), and dried in vacuo to afford white solids in excellent yields.

#### 3.2.2. General Procedure of the Metathesis Reaction

Bis[(trifluoromethyl)sulfonyl]imide (Tf_2_N) benzimidazole dicationic ionic liquids were obtained following a general procedure previously reported [12,35]. A water solution of lithium bis(trifuoromethylsulfonyl)imide (80% *w/w*, 1.2 equiv.) was added dropwise to an aqueous solution of opportune bromide benzimidazole dicationic ionic liquid (1 equiv., 10 mL). The suspension was stirred for 12 h at room temperature, and then 15 mL of dichloromethane was added. The organic phase was separated and washed with deionized water (3 × 15 mL) until bromide could not be detected (AgNO_3_ test). The organic phase was dried over Na_2_SO_4_, filtered, and evaporated under reduced pressure to provide a white solid in quantitative yields.

#### 3.2.3. 3,3’-(Propane-1,3-diyl)bis(1-methyl-1*H*-benzimidazolium) Dibromide C_3_(C_1_BenzIm)_2_/2Br

The synthesis of C_3_(C_1_BenzIm)_2_/2Br (1, 97% yield, white solid) was performed according to the general procedure described above for the preparation of the bromide BDILs. ^1^H NMR (CD_3_OD, after 2 h) δ 9.69 (s, 2H, 2 × H-2), 8.13–8.08 (m, 2H, Ar-H), 7.99–7.94 (m, 2H, Ar-H), 7.77–7.70 (m, 4H, Ar-H), 4.82 (t, 4H, J_vic_ = 7.4 Hz, 2 × CH_2_N), 4.16 (s, 6H, 2 × CH_3_N), 2.84–2.79 (m, 2H, CH_2_); ^13^C NMR (CD_3_OD, after 24 h) δ 143.4 (t, J = 33.5 Hz, 2 × C-2), 133.7, 132.7 (4 × Ar-C), 128.3 (4 × Ar-CH), 114.5 (4 × Ar-CH), 45.4 (2 × CH_2_N), 34.0 (2 × CH_3_N), 30.0 (CH_2_). NMR data (^1^H and ^13^C) were in agreement with those reported in DMSO-*d*_6_ [87,107].

#### 3.2.4. 3,3’-(Butane-1,4-diyl)bis(1-methyl-1*H*-benzimidazolium) Dibromide C_4_(C_1_BenzIm)_2_/2Br (**2**)

The synthesis of C_4_(C_1_BenzIm)_2_/2Br (**2**, 98% yield, white solid) was performed according to the general procedure described above for the preparation of the bromide BDILs. ^1^H NMR (CD_3_OD, after 20 min) δ 9.62 (s, 2H, 2 × H-2), 8.07–8.01 (m, 2H, Ar-*H*), 7.98–7.91 (m, 2H, Ar-*H*), 7.75–7.68 (m, 4H, Ar-*H*), 4.67–4.60 (m, 4H, 2 × C*H_2_*N), 4.16 (s, 6H, 2 × C*H_3_*N), 2.24–2.15 (m, 4H, 2 × C*H_2_*CH_2_N); ^13^C NMR (CD_3_OD, after 2 h) δ 143.5 (2 × C-2), 133.7, 132.7 (4 × Ar-*C*), 128.2, 128.2 (4 × Ar-*C*H), 114.5, 114.4 (4 × Ar-*C*H), 47.7 (2 × *C*H_2_N), 34.0 (2 × *C*H_3_N), 27.3 (2 × *C*H_2_CH_2_N). NMR data (^1^H and ^13^C) were in agreement with those reported in DMSO-d_6_ [87].

#### 3.2.5. 3,3’-(Pentane-1,5-diyl)bis(1-methyl-1*H*-benzimidazolium) Dibromide C_5_(C_1_BenzIm)_2_/2Br (**3**)

The synthesis of C_5_(C_1_BenzIm)_2_/2Br (**3**, 98% yield, white solid) was performed according to the general procedure described above for the preparation of the bromide BDILs. ^1^H NMR (CD_3_OD, after 10 min) δ 9.63 (s, 2H, 2 × H-2), 8.04–7.99 (m, 2H, Ar-*H*), 7.98–7.94 (m, 2H, Ar-*H*), 7.75–7.68 (m, 4H, Ar-*H*), 4.59 (t, 4H, *J*_vic_ = 7.3 Hz, 2 × C*H*_2_N), 4.17 (s, 6H, 2 × C*H_3_*N), 2.17–2.10 (m, 4H, 2 × C*H_2_*CH_2_N), 1.64–1.56 (m, 2H, C*H_2_*); ^13^C NMR (CD_3_OD, after 24 h) δ 143.2 (t, *J* = 31.4 Hz, 2 × C-2), 133.7, 132.8 (4 × Ar-*C*), 128.2 (4 × Ar-*C*H), 114.45, 114.4 (4 × Ar-*C*H), 48.3 (2 × *C*H_2_N), 33.9 (2 × *C*H_3_N), 30.0 (2 × *C*H_2_CH_2_N), 26.9 (*C*H_2_).

#### 3.2.6. 3,3’-(Hexane-1,6-diyl)bis(1-methyl-1*H*-benzimidazolium) Dibromide C_6_(C_1_BenzIm)_2_/2Br (**4**)

The synthesis of C_6_(C_1_BenzIm)_2_/2Br (**4**, 97% yield, white solid) was performed according to the general procedure described above for the preparation of the bromide BDILs. ^1^H NMR (CD_3_OD, after 10 min) δ 9.60 (s, 2H, 2 × H-2), 8.02–7.99 (m, 2H, Ar-*H*), 7.97–7.94 (m, 2H, Ar-*H*), 7.74–7.71 (m, 4H, Ar-*H*), 4.56 (t, 4H, *J*_vic_ = 7.3 Hz, 2 × C*H*_2_N), 4.16 (s, 6H, 2 × C*H_3_*N), 2.08–2.04 (m, 4H, 2 × C*H_2_*CH_2_N), 1.57–1.53 (m, 4H, C*H_2_*C*H_2_*); ^13^C NMR (CD_3_OD, after 24 h) δ 143.1 (t, *J* = 33.10 Hz, 2 × C-2), 133.7, 132.8 (4 × Ar-*C*), 128.2 (4 × Ar-*C*H), 114.4, 114.3 (4 × Ar-*C*H), 48.2 (2 × *C*H_2_N), 33.9 (2 × *C*H_3_N), 30.0 (2 × *C*H_2_CH_2_N), 26.9 (*C*H_2_*C*H_2_).

#### 3.2.7. 3,3’-(Propane-1,3-diyl)bis(1-methyl-1*H*-benzimidazolium) diBis[(Trifluoromethyl)Sulfonyl]Imide C_3_(C_1_BenzIm)_2_/2Tf_2_N (**5**)

The synthesis of C_3_(C_1_BenzIm)_2_/2Br (**5**, 99% yield, white solid) was performed according to the general procedure described above for the preparation of the bis[(trifluoromethyl)sulfonyl]imide BDILs. ^1^H NMR (CD_3_OD, after 2 h) δ 9.39 (s, 2H, 2 × H-2), 8.01–7.98 (m, 2H, Ar-*H*), 7.97–7.93 (m, 2H, Ar-*H*), 7.77–7.71 (m, 4H, Ar-*H*), 4.73 (t, 4H, *J*_vic_ = 7.5 Hz, 2 × C*H_2_*N), 4.13 (s, 6H, 2 × C*H_3_*N), 2.80–2.75 (m, 2H, C*H_2_*); ^13^C NMR (CD_3_OD, after 24 h) δ 143.1 (t, *J* = 32.0 Hz, 2 × C-2), 133.7, 132.6 (4 × Ar-*C*), 128.5 (4 × Ar-*C*H), 121.2 (q, *J*_C,F_ = 320.7 Hz, 4 × CF_3_), 114.5, 114.2 (4 × Ar-*C*H), 45.3 (2 × *C*H_2_N), 33.9 (2 × *C*H_3_N), 29.9 (*C*H_2_).

#### 3.2.8. 3,3’-(Butane-1,4-diyl)bis(1-methyl-1*H*-benzimidazolium) diBis[(Trifluoromethyl)Sulfonyl]Imide C_4_(C_1_BenzIm)_2_/2Tf_2_N (**6**)

The synthesis of C_4_(C_1_BenzIm)_2_/2Tf_2_N (**6**, 99% yield, white solid) was performed according to the general procedure described above for the preparation of the bis[(trifluoromethyl)sulfonyl]imide BDILs. ^1^H NMR (CD_3_OD, after 2 h) δ 9.62 (s, 2H, 2 × H-2), 7.99–7.92 (m, 4H, Ar-*H*), 7.75–7.68 (m, 4H, Ar-*H*), 4.61–4.58 (m, 4H, 2 × C*H_2_*N), 4.12 (s, 6H, 2 × C*H_3_*N), 2.17–2.14 (m, 4H, 2 × C*H_2_*CH_2_N); ^13^C NMR (CD_3_OD, after 24 h) δ 143.0 (t, *J* = 32.5 Hz, 2 × C-2), 133.7, 132.7 (4 × Ar-*C*), 128.3 (4 × Ar-*C*H), 121.1 (q, *J*_C,F_ = 320.7 Hz, 4 × CF_3_), 114.4, 114.3 (4 × Ar-*C*H), 47.7 (2 × *C*H_2_N), 33.8 (2 × *C*H_3_N), 27.2 (2 × *C*H_2_CH_2_N).

#### 3.2.9. 3,3’-(Pentane-1,5-diyl)bis(1-methyl-1*H*-benzimidazolium) diBis[(Trifluoromethyl)Sulfonyl]Imide C_5_(C_1_BenzIm)_2_/2Tf_2_N (**7**)

The synthesis of C_5_(C_1_BenzIm)_2_/2Tf_2_N (**7**, 99% yield, white solid) was performed according to the general procedure described above for the preparation of the bis[(trifluoromethyl)sulfonyl]imide BDILs. ^1^H NMR (CD_3_OD, after 2 h) δ 9.29 (s, 2H, 2 × H-2), 7.93–7.87 (m, 4H, Ar-*H*), 7.04–7.64 (m, 4H, Ar-*H*), 4.51 (t, 4H, *J*_vic_ = 7.4 Hz, 2 × C*H*_2_N), 4.10 (s, 6H, 2 × C*H_3_*N), 2.14–2.06 (m, 4H, 2 × C*H_2_*CH_2_N), 1.61–1.53 (m, 2H, C*H_2_*); ^13^C NMR (CD_3_OD, after 24 h) δ 142.7 (t, *J* = 33.3 Hz, 2 × C-2), 133.5, 132.7 (4 × Ar-*C*), 128.1 (4 × Ar-*C*H), 121.1 (q, *J*_C,F_ = 320.6 Hz, 4 × CF_3_), 114.2 (4 × Ar-*C*H), 48.0 (2 × *C*H_2_N), 33.8 (2 × *C*H_3_N), 29.7 (2 × *C*H_2_CH_2_N), 24.4 (*C*H_2_).

#### 3.2.10. 3,3’-(Hexane-1,5-diyl)bis(1-methyl-1*H*-benzimidazolium) diBis[(Trifluoromethyl)Sulfonyl]Imide C_6_(C_1_BenzIm)_2_/2Tf_2_N (**8**)

The synthesis of C_6_(C_1_BenzIm)_2_/2Tf_2_N (**8**, 99% yield, white solid) was performed according to the general procedure described above for the preparation of the bis[(trifluoromethyl)sulfonyl]imide BDILs. ^1^H NMR (CD_3_OD, after 2 h) δ 9.34 (s, 2H, 2 × H-2), 7.96–7.90 (m, 4H, Ar-*H*), 7.73–7.67 (m, 4H, Ar-*H*), 4.51 (t, 4H, *J*_vic_ = 7.3 Hz, 2×C*H*_2_N), 4.12 (s, 6H, 2 × C*H_3_*N), 2.08–2.00 (m, 4H, 2 × C*H_2_*CH_2_N), 1.55–1.51 (m, 4H, C*H_2_*C*H_2_*); ^13^C NMR (CD_3_OD, after 24 h) δ 143.8 (t, *J* = 32.6 Hz, 2 × C-2), 133.6, 132.8 (4 × Ar-*C*), 128.2 (4 × Ar-*C*H), 121.2 (q, *J*_C,F_ = 322.6 Hz, 4 × CF_3_), 114.3, 114.3 (4 × Ar-*C*H), 48.3 (2 × *C*H_2_N), 33.7 (2 × *C*H_3_N), 30.0 (2 × *C*H_2_CH_2_N), 26.9 (*C*H_2_*C*H_2_).

### 3.3. Thermal Analysis

Prior to TGA and DSC measurement, all samples were dried in high vacuum at 60 °C for 24 h to remove moisture.

#### 3.3.1. Thermal Gravimetric Analysis

The thermal stability of the synthesized ILs was investigated by using thermal gravimetric analysis (TA Instruments Q500 TGA, New Castle, DE, USA). The instrument was calibrated using weight standards (1 g and 100 mg). The temperature calibration was performed using nickel standards. All the standards were supplied by TA Instruments Inc (New Castle, DE, USA). The sample (10–15 mg) was heated at 60 °C in a platinum crucible for the drying procedure and maintained in N_2_ flux (90 mL/min) for 30 min. Then, BDIL was heated from 40 to 700 °C with a heating rate of 10 °C/min under nitrogen (90 mL/min) and maintained at 700 °C for 3 min. The mass change was recorded as a function of temperature and time. TGA experiments were carried out in duplicate.

#### 3.3.2. Differential Scanning Calorimetry

The thermal behavior of the ionic liquids was analyzed by using a differential scanning calorimeter (TA DSC Q250, New Castle, DE, USA). The temperature calibration was performed considering the heating rate dependence of the onset temperature of the melting peak of indium. In order to allow volatile compounds to escape and to avoid the risk of the pan bursting under pressure, a small incision was made at the top of both the sample and the reference pan. The sample (3–5 mg) was loaded in Tzero hermetic aluminum pan (TA Instruments), and the phase behavior was explored under nitrogen atmosphere (30 mL/min) in the temperature range of −90 to 200 °C at different scanning rates of (2–10 °C/min). DSC experiments were carried out in duplicate. *T*_g_ was obtained by taking the onset of the heat capacity change on heating from a glass to a liquid. *T*_cc_ was determined as the peak temperature of the exothermic peak on heating from a subcooled liquid to a crystalline solid. *T*_m_ was taken as the peak temperature of the endothermic peak on the heating run. The peak temperatures were chosen instead of the onset temperatures due to the complexity of the thermograms.

#### 3.3.3. Cyclic Voltammetry

Cyclic voltammetry (CV) experiments were conducted at room temperature under N_2_ on previously deaerated samples, in a potential range between −2.00 and 3.20 V (vs. Ag/AgCl) at 100 mVs^−1^ potential scan rate. Electrochemical stability windows (ECW) have been reported considering the cathodic and anodic limits as the CV onset potentials.

## 4. Conclusions

A series of dicationic ionic liquids, based on the same methyl benzimidazole cationic core but bearing alkyl spacers of different lengths, were prepared and profiled. The ILs in question, belonging to two structurally related families of dicationic ionic liquids, feature C3 to C6 spacers between the benzimidazole cores and are characterized by either bromide or bistriflimide counter anions. The systematic study of their thermal properties and the cyclic voltammetry analysis elucidated the effect of the different anions and of the spacer length on their properties and behavior. While the thermogravimetric analysis only confirmed the expected higher stability of the bistriflimide ILs when compared to the bromide ILs, on account of the higher nucleophilicity of the halide anion, DSC analysis presented a more complex picture. Indeed, in the case of the bistriflimide series, all possible types of thermal behavior for ILs were found. Therefore, within this family, the distance between the cationic headgroups plays a dramatic effect on whether or not a phase transition may occur. This reflects in a different liquid range, which is the widest in the case of C_5_(C_1_BenzIm)_2_/2Tf_2_N. Again, cyclic voltammetry showed different electrochemical windows (C_3_(C_1_BenzIm)_2_/2Tf_2_N < C_4_(C_1_BenzIm)_2_/2Tf_2_N, C_5_(C_1_BenzIm)_2_/2Tf_2_N < C_6_(C_1_BenzIm)_2_/2Tf_2_N) as well as a reduction in peak potential, shape, and intensity as a function of the spacer length.

On the basis of the presented results, it would be of interest to investigate the effect of the linker length on the catalytic activities of the proposed BDILs in organic reactions and on the ability to furnish organic super-electron donors.

## Data Availability

Not applicable.

## Supplementary Materials

The following are available online, Figure S1. ^1^H NMR of compound 1, Figure S2. ^13^C NMR of compound 1, Figure S3. ^1^H NMR of compound 2, Figure S4. ^13^C NMR of compound 2, Figure S5. ^1^H NMR of compound 3 (after 10 min), Figure S6. ^1^H NMR of compound 3 (after 24 h), Figure S7. ^1^H NMR of compound 3 (after 10 min and 24 h), Figure S8. ^13^C NMR of compound 3, Figure S9. ^1^H NMR of compound 4 (after 15 min), Figure S10. ^1^H NMR of compound 4 (after 24 h), Figure S11. ^1^H NMR of compound 4 (after 15 min and 24 h), Figure S12. ^13^C NMR of compound 4 (after 2 h), Figure S13. ^13^C NMR of compound 4 (after 24 h), Figure S14. ^13^C NMR of compound 4 (after 2 h and 24 h), Figure S15. ^1^H NMR of compound 5, Figure S16. ^13^C NMR of compound 5, Figure S17. ^1^H NMR of compound 6, Figure S18. ^13^C NMR of compound 6, Figure S19. ^1^H NMR of compound 7, Figure S20. ^13^C NMR of compound 7, Figure S21. ^1^H NMR of compound 8, Figure S22. ^13^C NMR of compound 8, Figure S23. TGA of compound 1, Figure S24. TGA of compound 2, Figure S25. TGA of compound 3, Figure S26. TGA of compound 4, Figure S27. TGA of compound 5, Figure S28: TGA of compound 6, Figure S29 TGA of compound 7, Figure S30. TGA of compound 8, Figure S31. DSC of compound 1, Figure S32. DSC of compound 2, Figure S33. DSC of compound 3, Figure S34. DSC of compound 4, Figure S35. DSC of compound 5, Figure S36. DSC of compound 6, Figure S37. DSC of compound 7, Figure S38. DSC of compound 8, Figure S39. Cyclic voltammetry of compound 5, Figure S40. Cyclic voltammetry of compound 6, Figure S41. Cyclic voltammetry of compound 7, Figure S42. Cyclic voltammetry of compound 8, Table S1. T_start_, T_onset_, and T_peak_ of the investigated ionic liquids measured at heating rate of 10 °C/min, Table S2. Melting temperature (T_m_), and enthalpy (H_m_) of the investigated ionic liquids measured at first heating run, Table S3. Glass transition temperature (T_g_), crystallization temperature (T_c_), cold crystallization temperature (T_cc_), and melting temperature (T_m_) of the investigated ionic liquids measured at second heating run, Table S4. Apparent activation energy (E_a_), consequently its fragility index (m), and glass transition temperature (T_g_/K) of compounds 7.

## Sample Availability

Samples of the compounds are available from the authors.
