# Supplementary material for: Expanding the Chemical Space of Benzimidazole Dicationic Ionic Liquids
Source: Molecules. 2021 Jul 11;26(14):4211. doi: 10.3390/molecules26144211 (PMC8303995; doi:10.3390/molecules26144211)
Supplement: Supplementary file 1 [file molecules-26-04211-s001.zip › molecules-1284794-SI.pdf]

Expanding the Chemical Space of Benzimidazole Dicationic Ionic Liquids

Andrea Mezzetta [1,*], Luca Guglielmero [1,2], Angelica Mero [1], Giorgio Tofani [1,3], Felicia D'Andrea [1], Christian Silvio Pomelli [1] and Lorenzo Guazzelli [1]

[1] Department of Pharmacy, University of Pisa, Via Bonanno 33, 56126 Pisa, Italy; luca.guglielmero@phd.unipi.it (L.G.); angelica.mero@phd.unipi.it (A.M.); giorgio.tofani@df.unipi.it (G.T.); felicia.dandrea@farm.unipi.it (F.D.); christian.pomelli@unipi.it (C.S.P.); lorenzo.guazzelli@unipi.it (L.G.)
[2] DESTEC, University of Pisa, Largo Lucio Lazzarino, 56122 Pisa, Italy
[3] Department of Physics, University of Pisa, Largo Bruno Pontecorvo 3, 56127 Pisa, Italy
* Correspondence: andrea.mezzetta@unipi.it

Supporting Information

Table of contents

^{1}H and ^{13}C NMR of compound **1**–**8** pages S2–S23

Thermal gravimetric analysis (TGA) of compounds **1**–**8** pages S24–S32

Differential scanning calorimetry (DSC) of compounds **1**–**8** pages S33–S42

Cyclic voltammetry of compounds **5**–**8** pages S43–S46

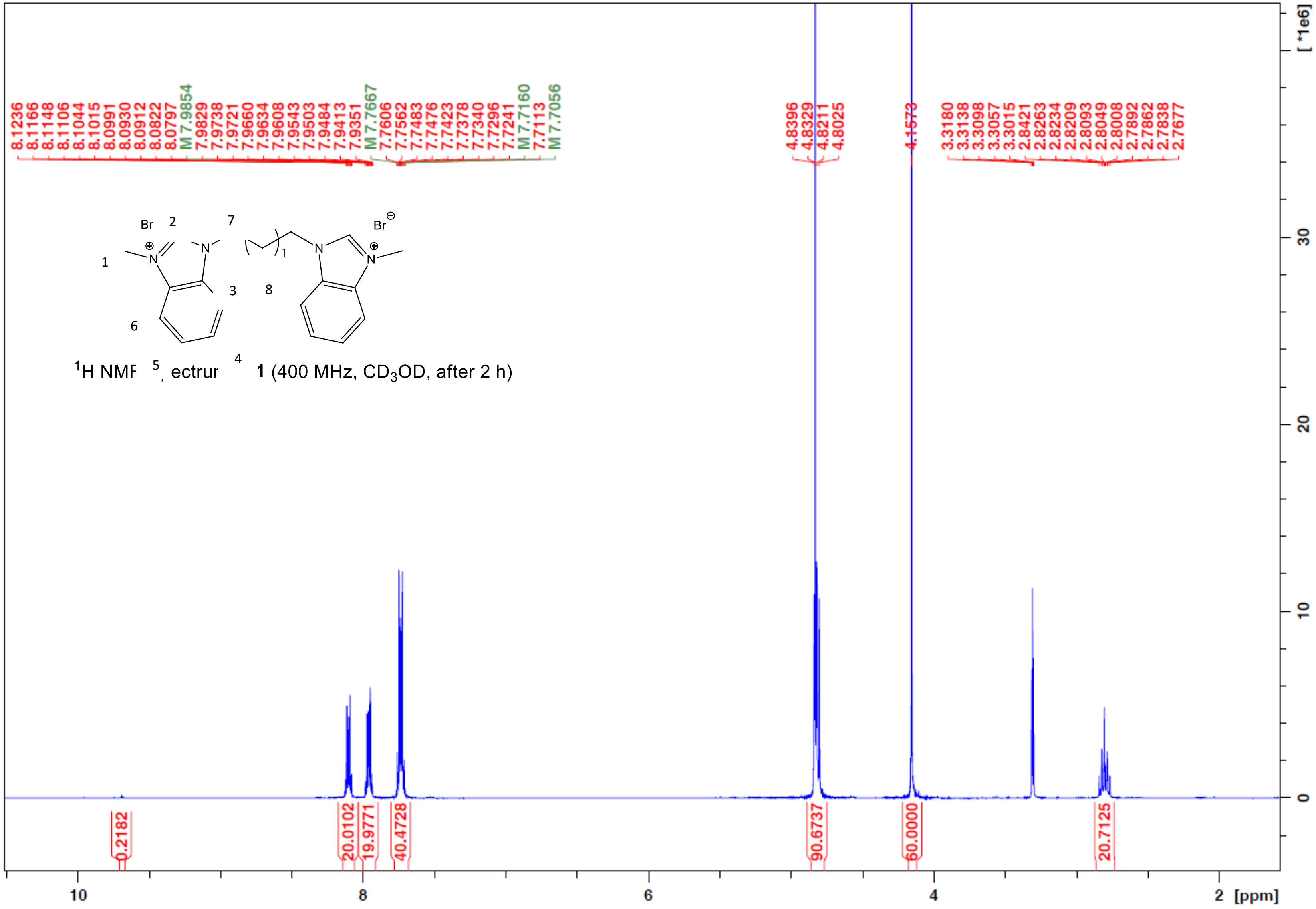

Figure S1. ¹H NMR of compound **1**.

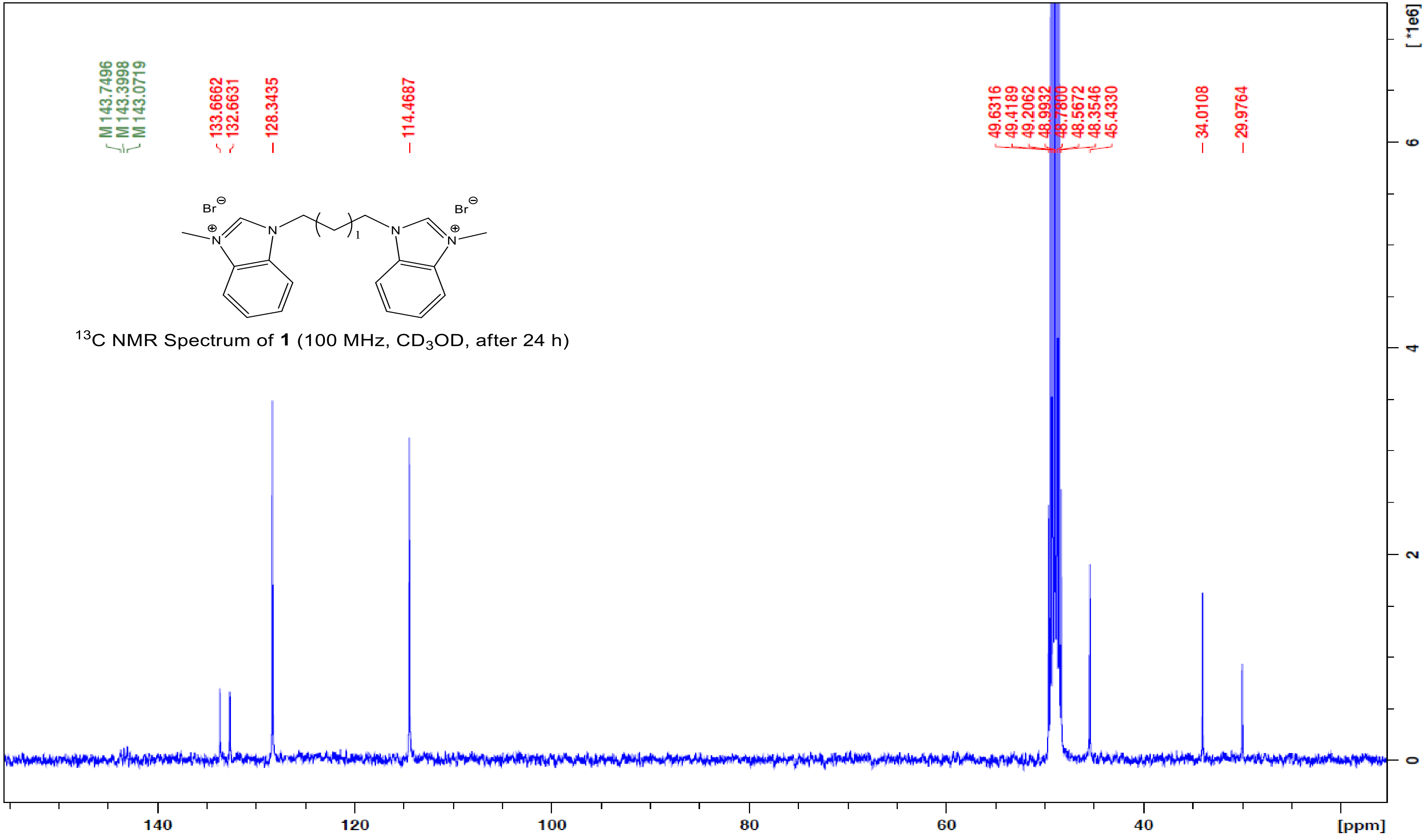

Figure S2. ^{13}C NMR of compound **1**.

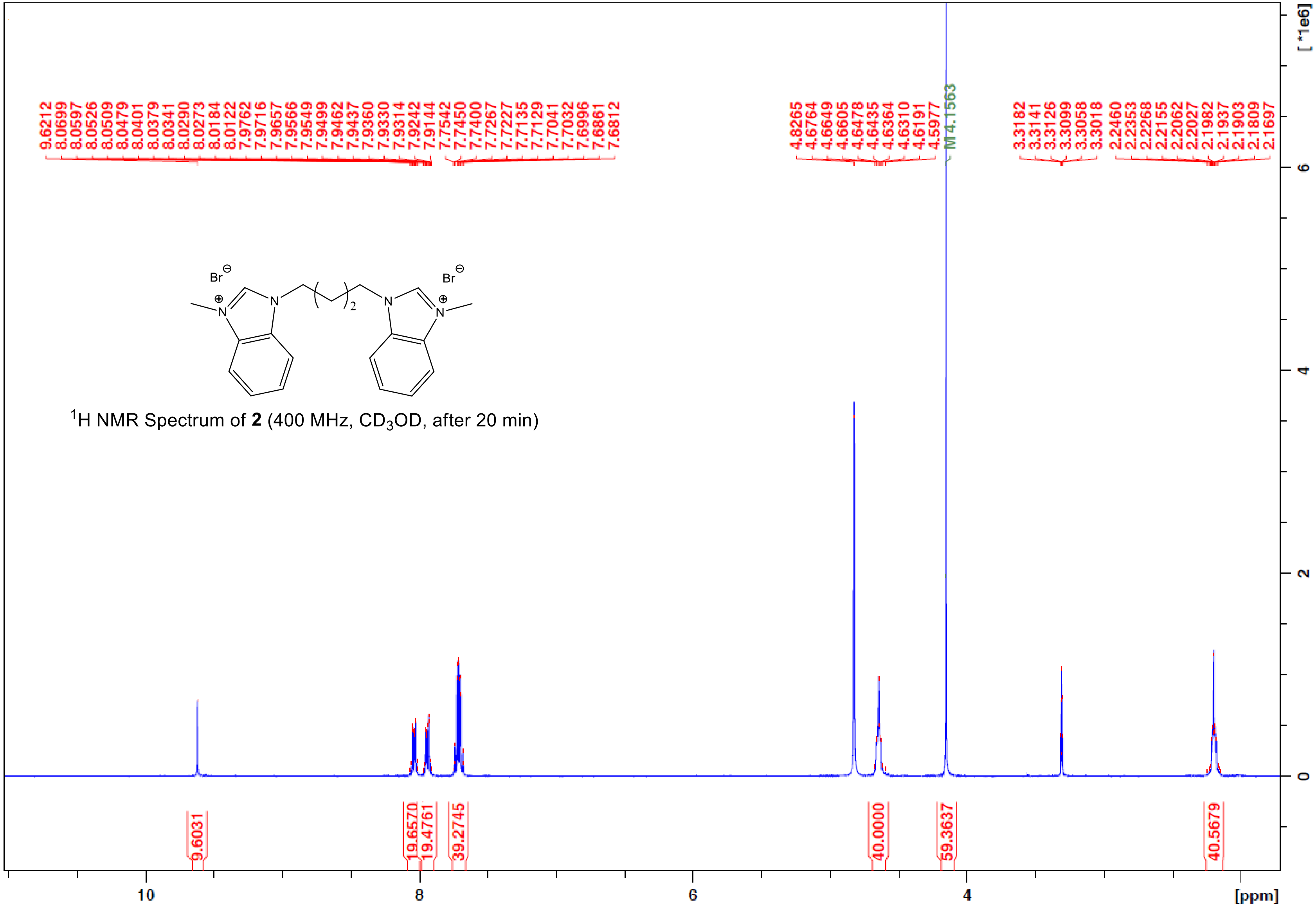

^{1}H NMR Spectrum of **2** (400 MHz, CD_3OD, after 20 min)

Figure S3. ^{1}H NMR of compound **2**.

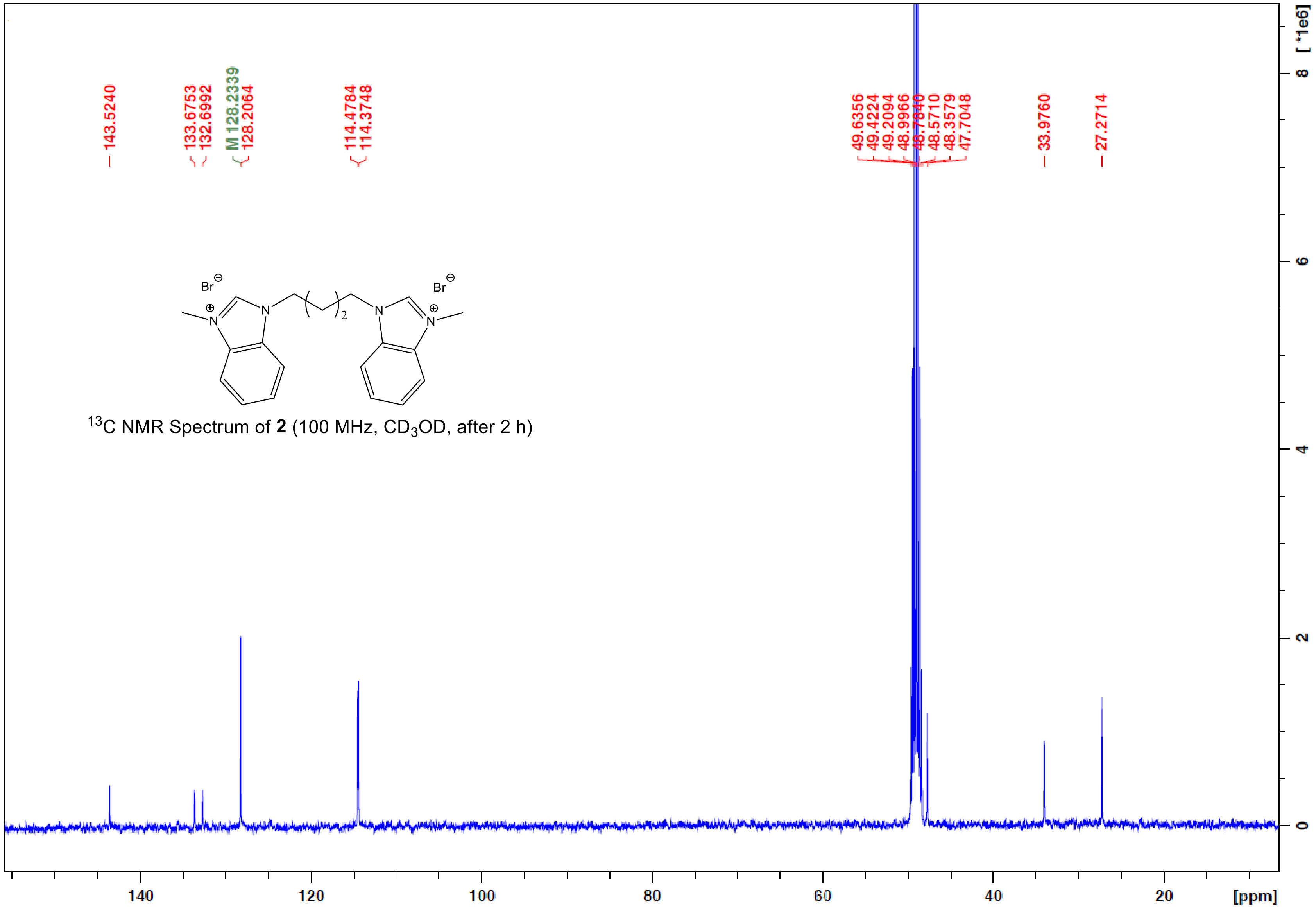

Figure S4. ^{13}C NMR of compound **2**.

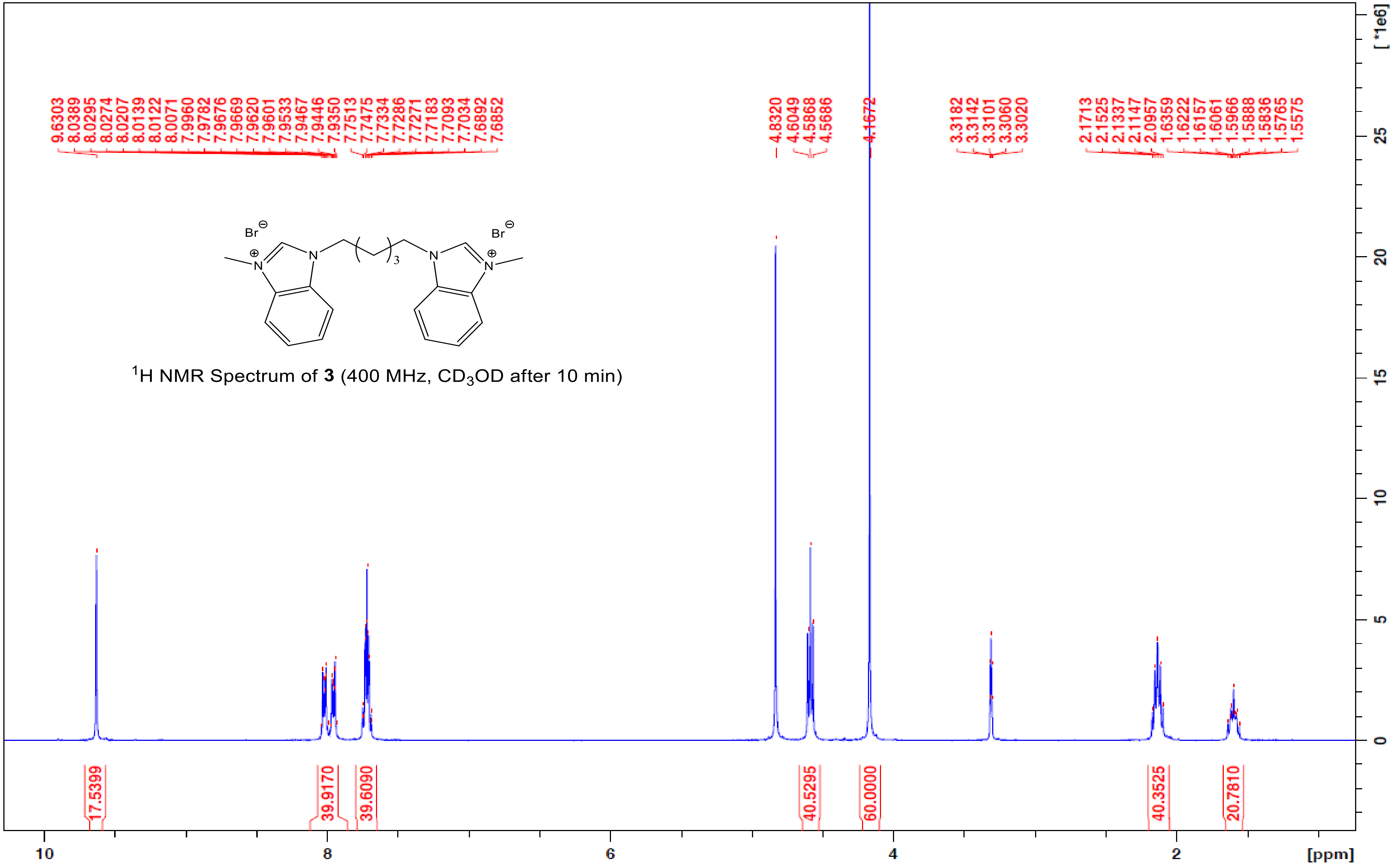

^{1}H NMR Spectrum of **3** (400 MHz, CD_3OD after 10 min)

Figure S5. ^{1}H NMR of compound **3** (after 10 min).

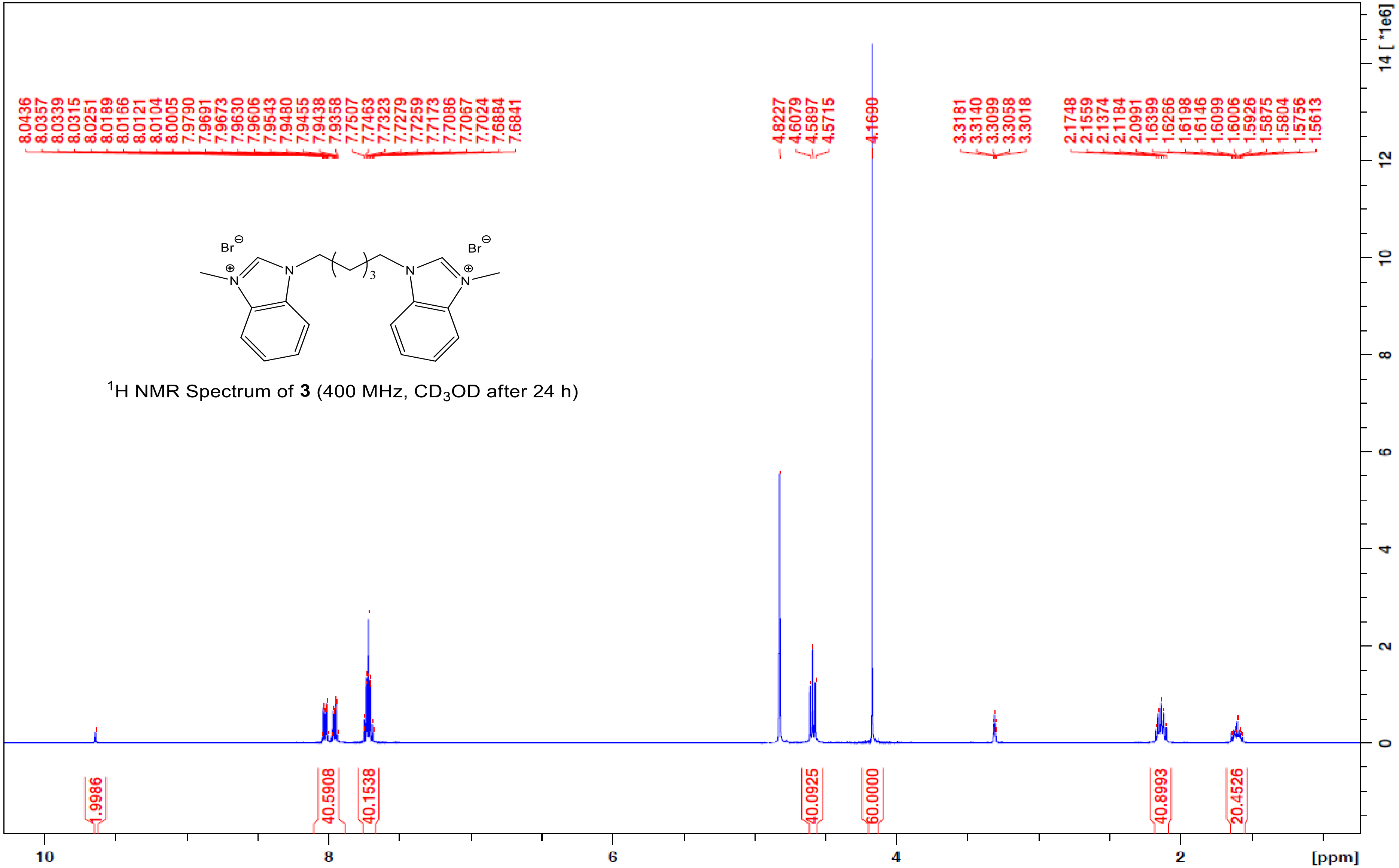

^{1}H NMR Spectrum of **3** (400 MHz, CD_3OD after 24 h)

Figure S6. ^{1}H NMR of compound **3** (after 24 h)

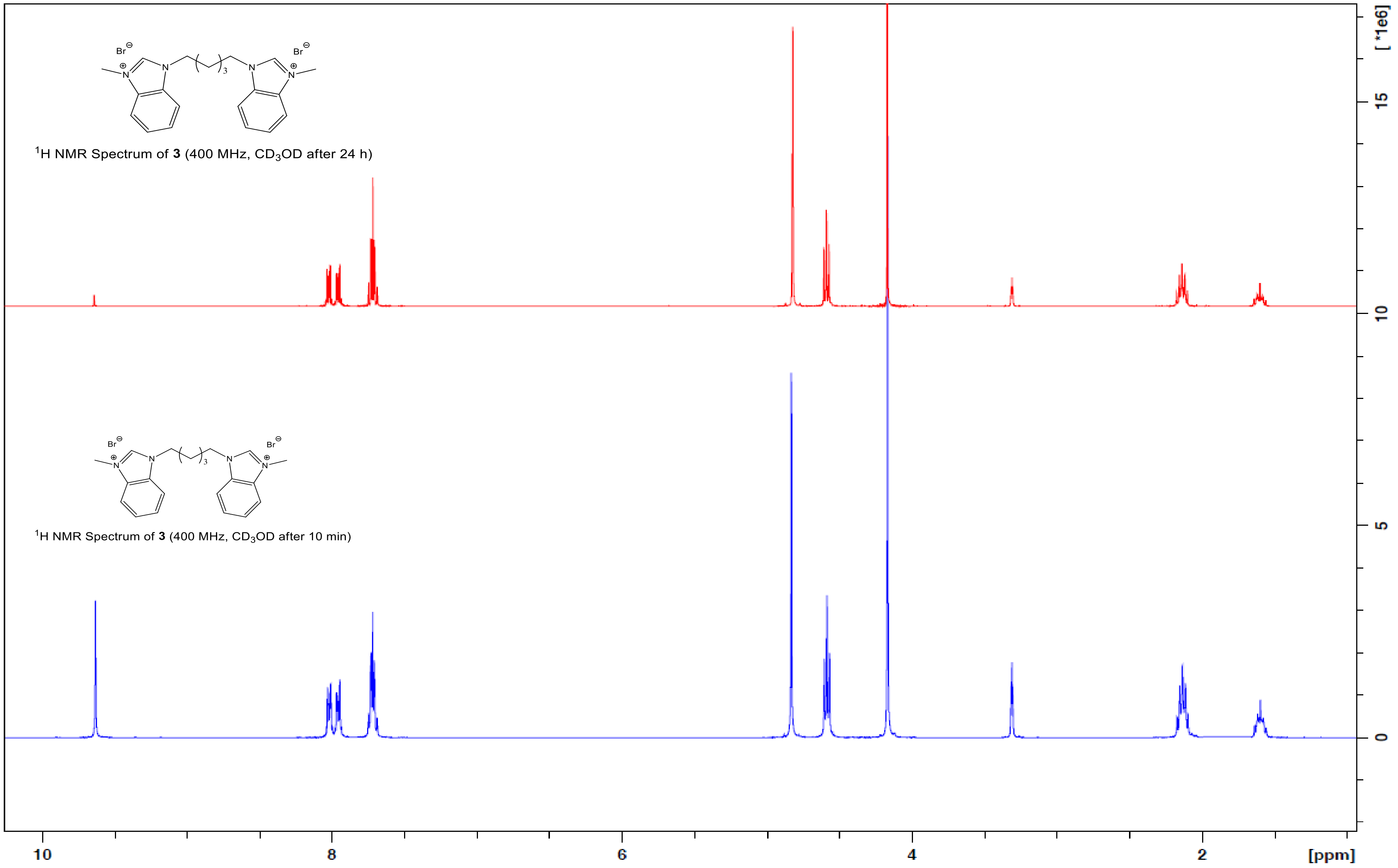
^{1}H NMR Spectrum of **3** (400 MHz, CD_3OD after 24 h)
^{1}H NMR Spectrum of **3** (400 MHz, CD_3OD after 10 min)
[*1e6]
15
10
5
0
10
8
6
4
2
[ppm]

Figure S7. ^{1}H NMR of compound **3** (after 10 min and 24 h)

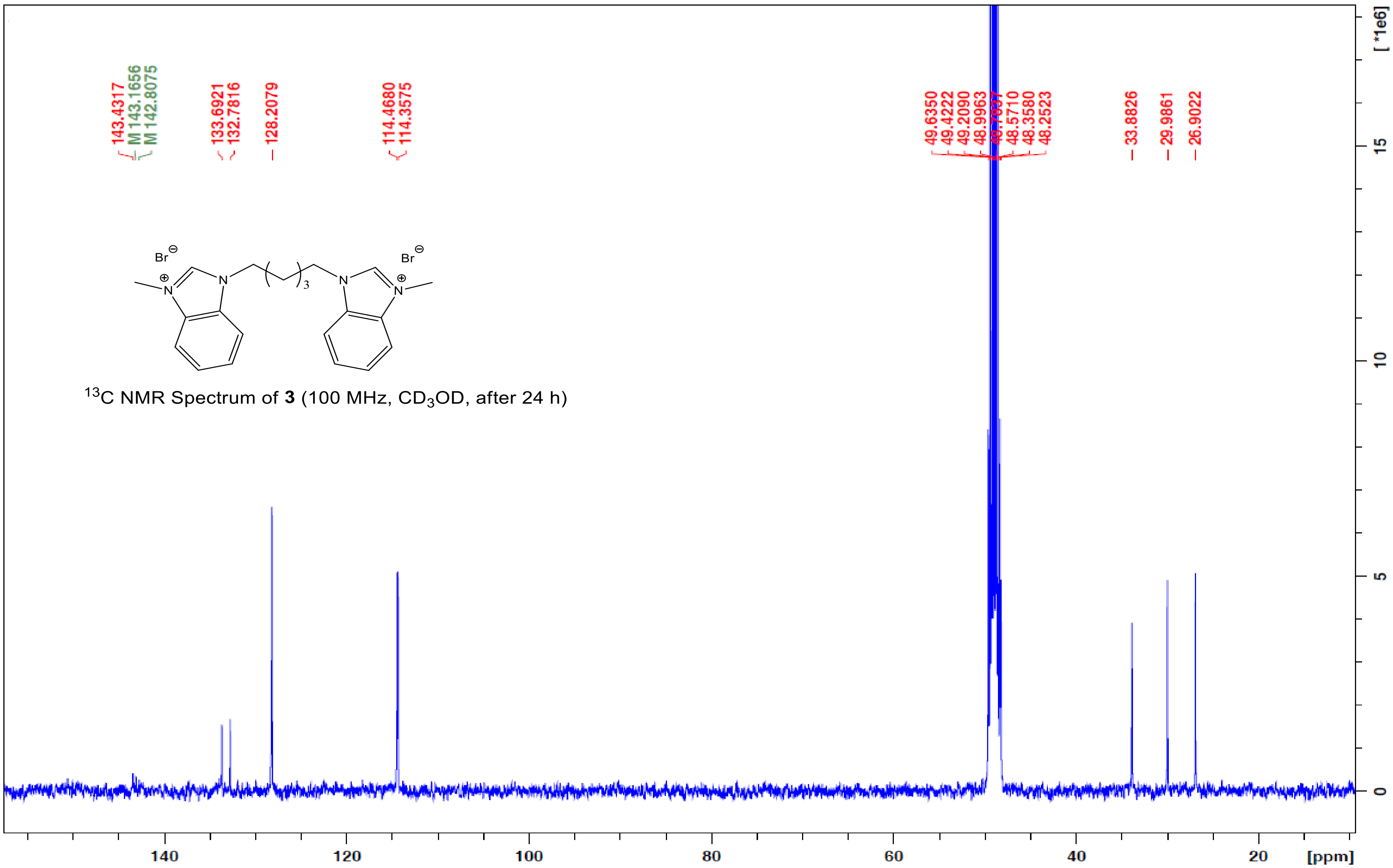

Figure S6. ^{13}C NMR of compound **3**.

Figure S8. ^{13}C NMR of compound **3**.

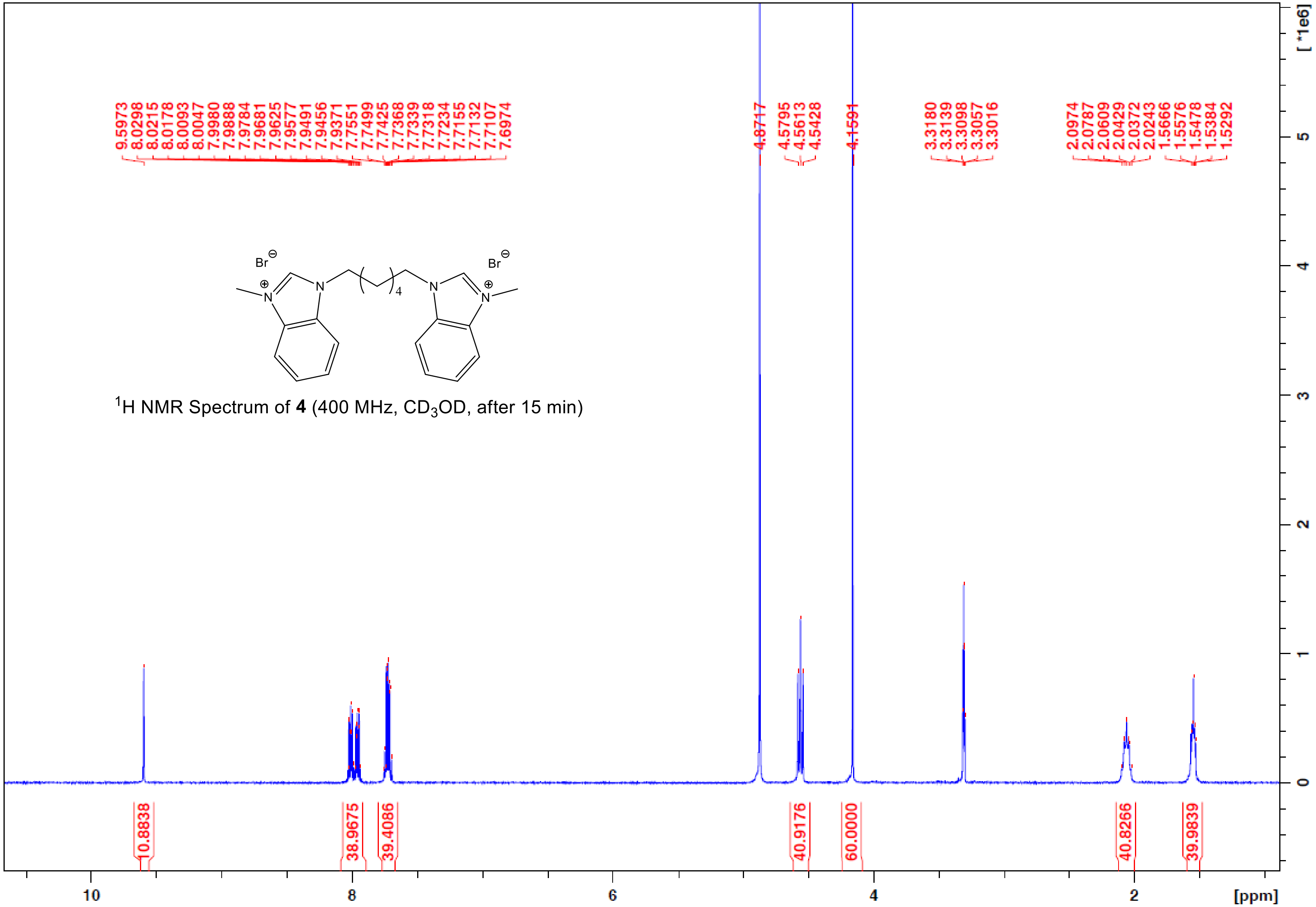

^{1}H NMR Spectrum of **4** (400 MHz, CD_3OD, after 15 min)

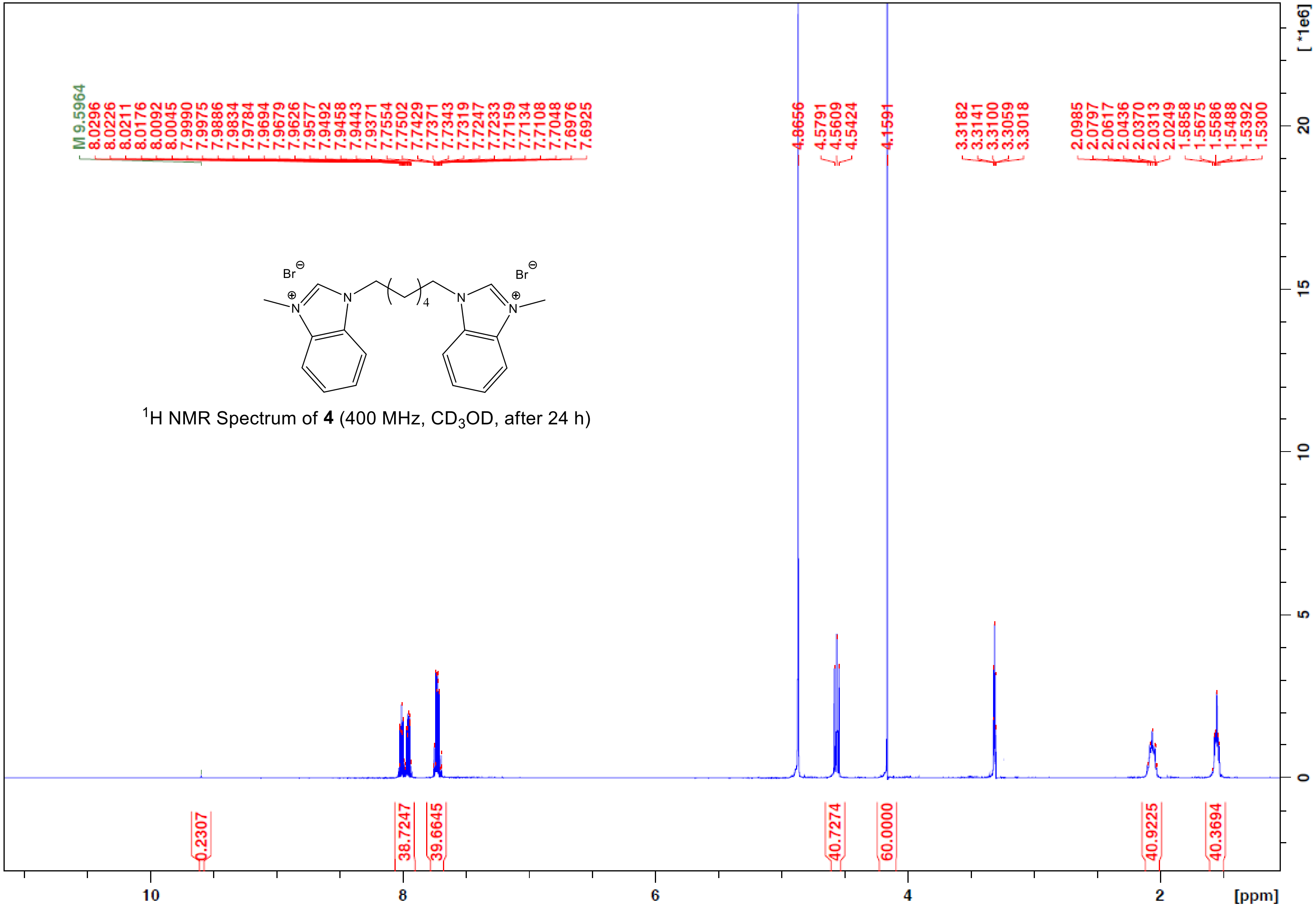

^{1}H NMR Spectrum of **4** (400 MHz, CD_3OD, after 24 h)

Figure S10. ^{1}H NMR of compound **4** (after 24 h)

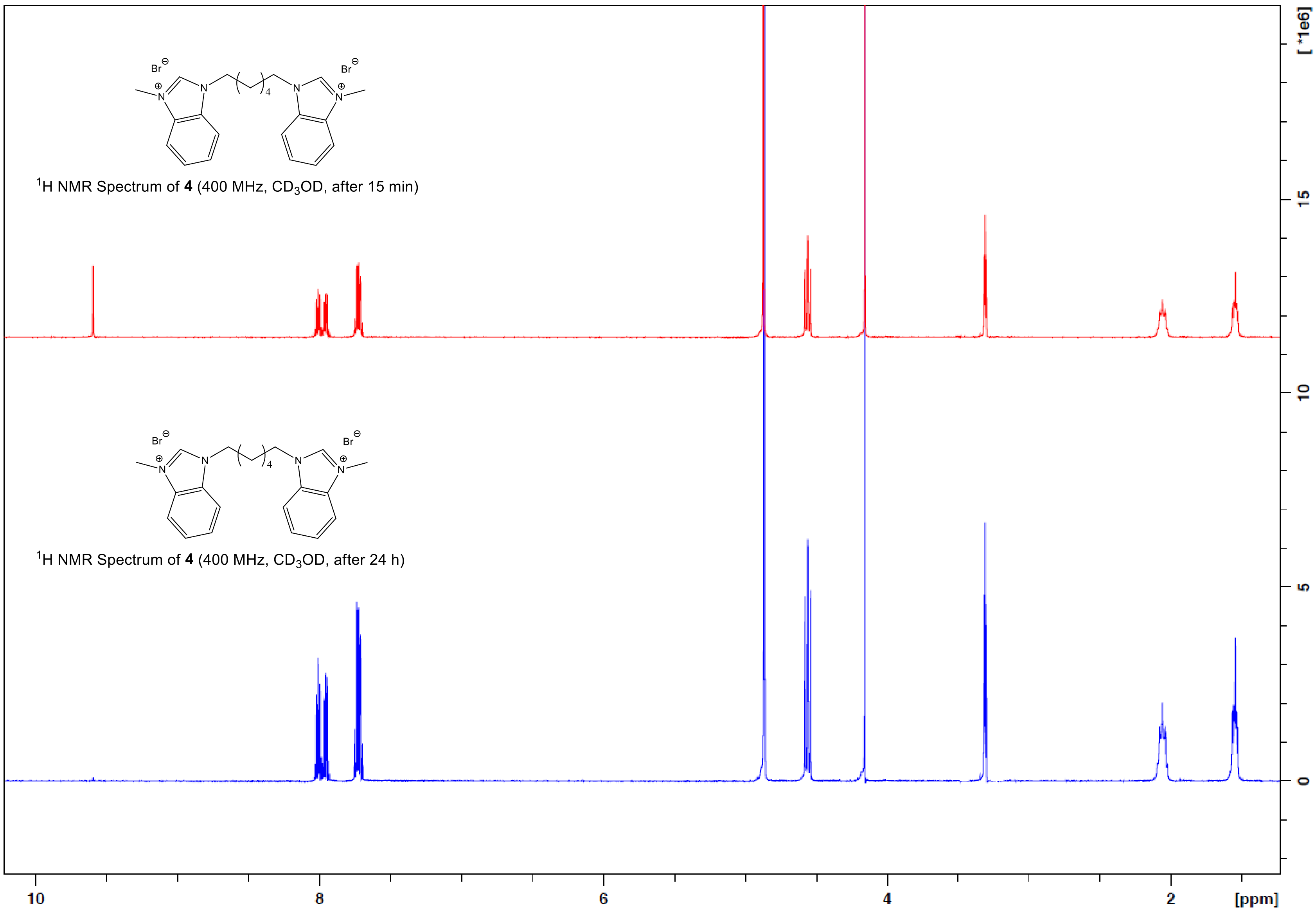
Br⊖
Br⊖
N
N
4
1H NMR Spectrum of 4 (400 MHz, CD3OD, after 15 min)
1H NMR Spectrum of 4 (400 MHz, CD3OD, after 24 h)
[*1e6]
15
10
5
0
10
8
6
4
2
[ppm]

Figure S11. ^{1}H NMR of compound **4** (after 15 min and 24 h)

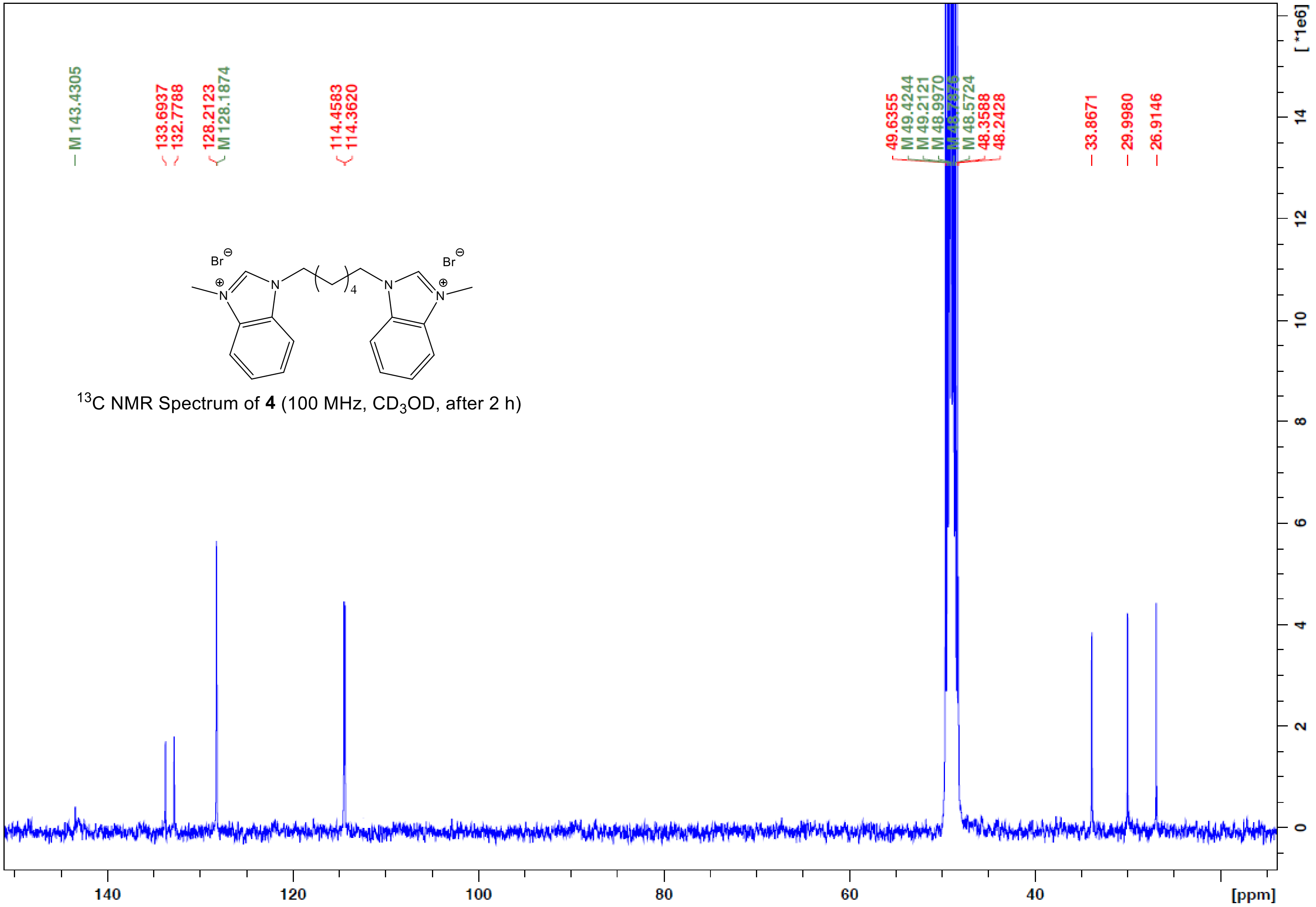

^{13}C NMR Spectrum of **4** (100 MHz, CD_3OD, after 2 h)

Figure S12. ^{13}C NMR of compound **4** (after 2 h)

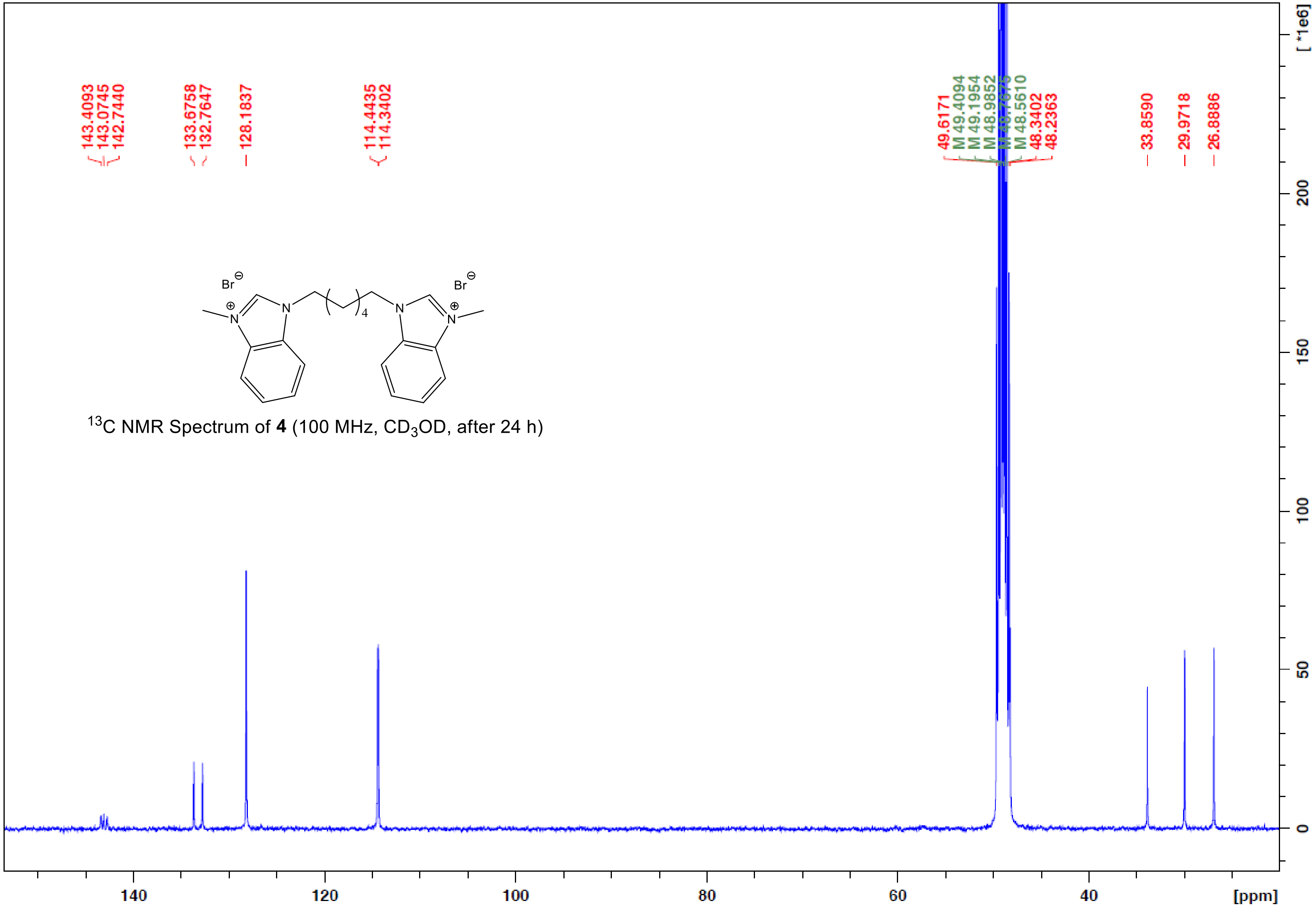

^{13}C NMR Spectrum of **4** (100 MHz, CD_3OD, after 24 h)

Figure S13. ^{13}C NMR of compound **4** (after 24 h)

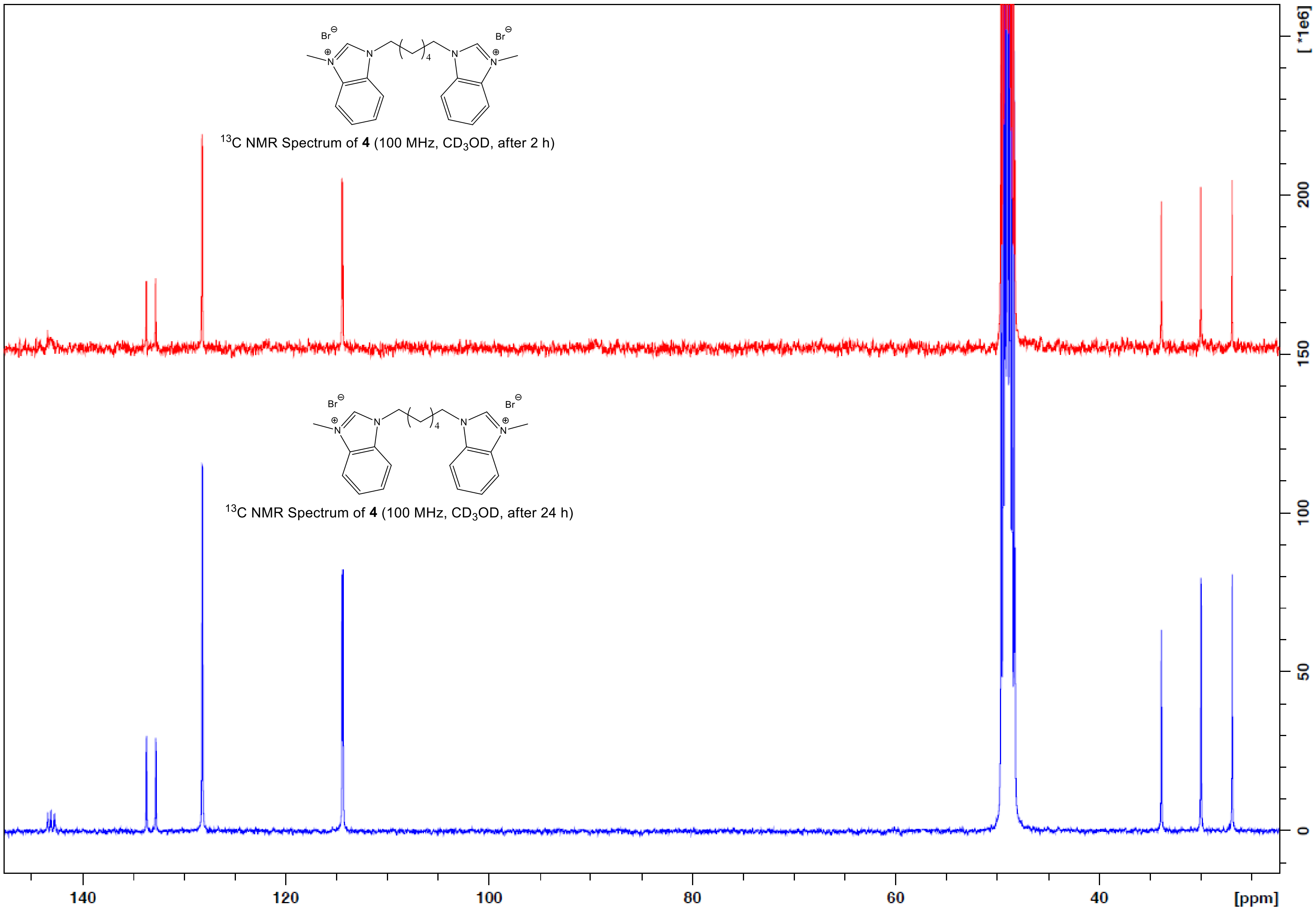

Br⊖
Br⊖
⊕N
N
N
N⊕
4
^{13}C NMR Spectrum of **4** (100 MHz, CD_3OD, after 2 h)
^{13}C NMR Spectrum of **4** (100 MHz, CD_3OD, after 24 h)
[*1e6]
200
150
100
50
0
140
120
100
80
60
40
[ppm]

Figure S14. ^{13}C NMR of compound **4** (after 2 h and 24 h)

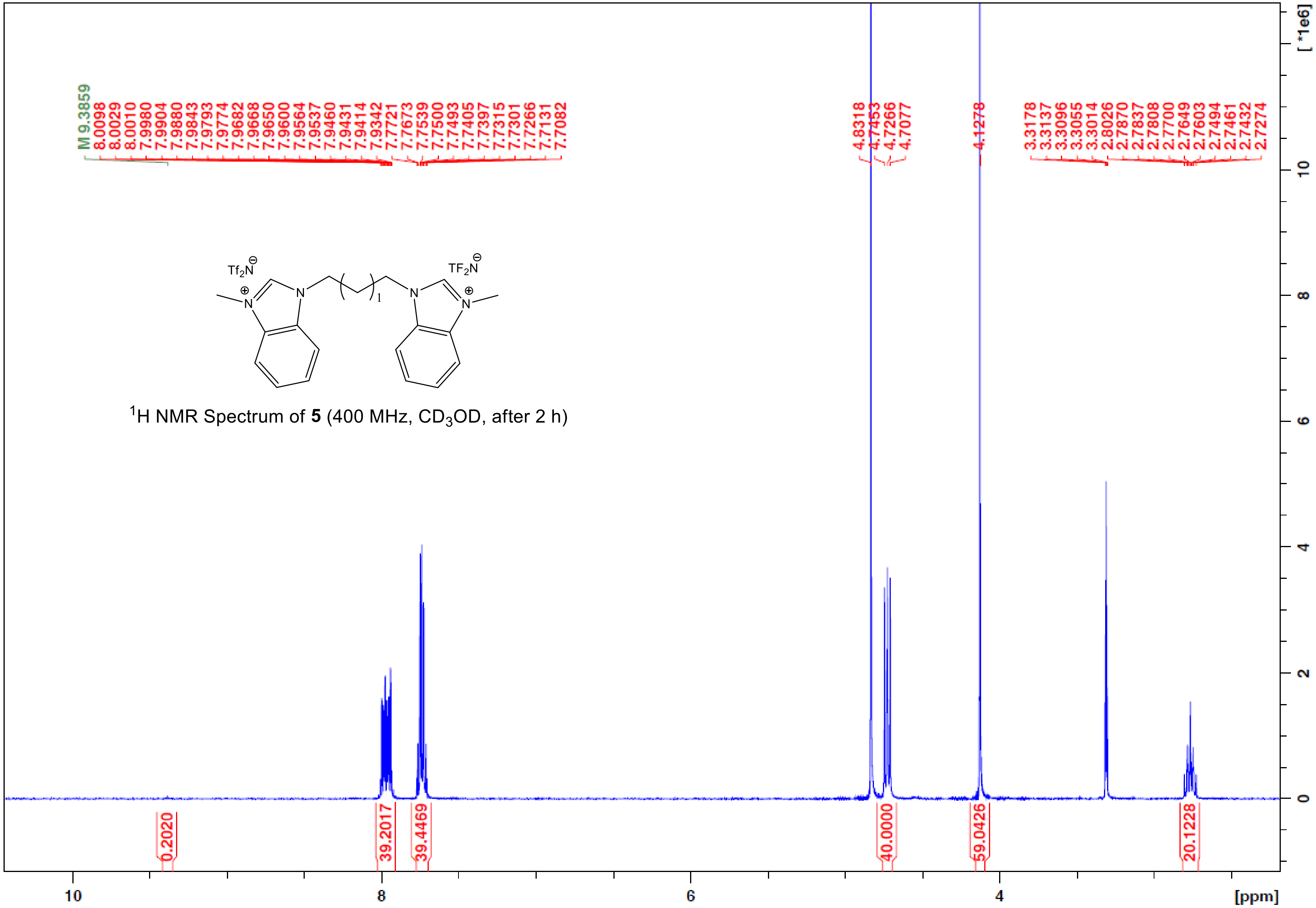

^{1}H NMR Spectrum of **5** (400 MHz, CD_3OD, after 2 h)

Figure S15. ^{1}H NMR of compound **5**.

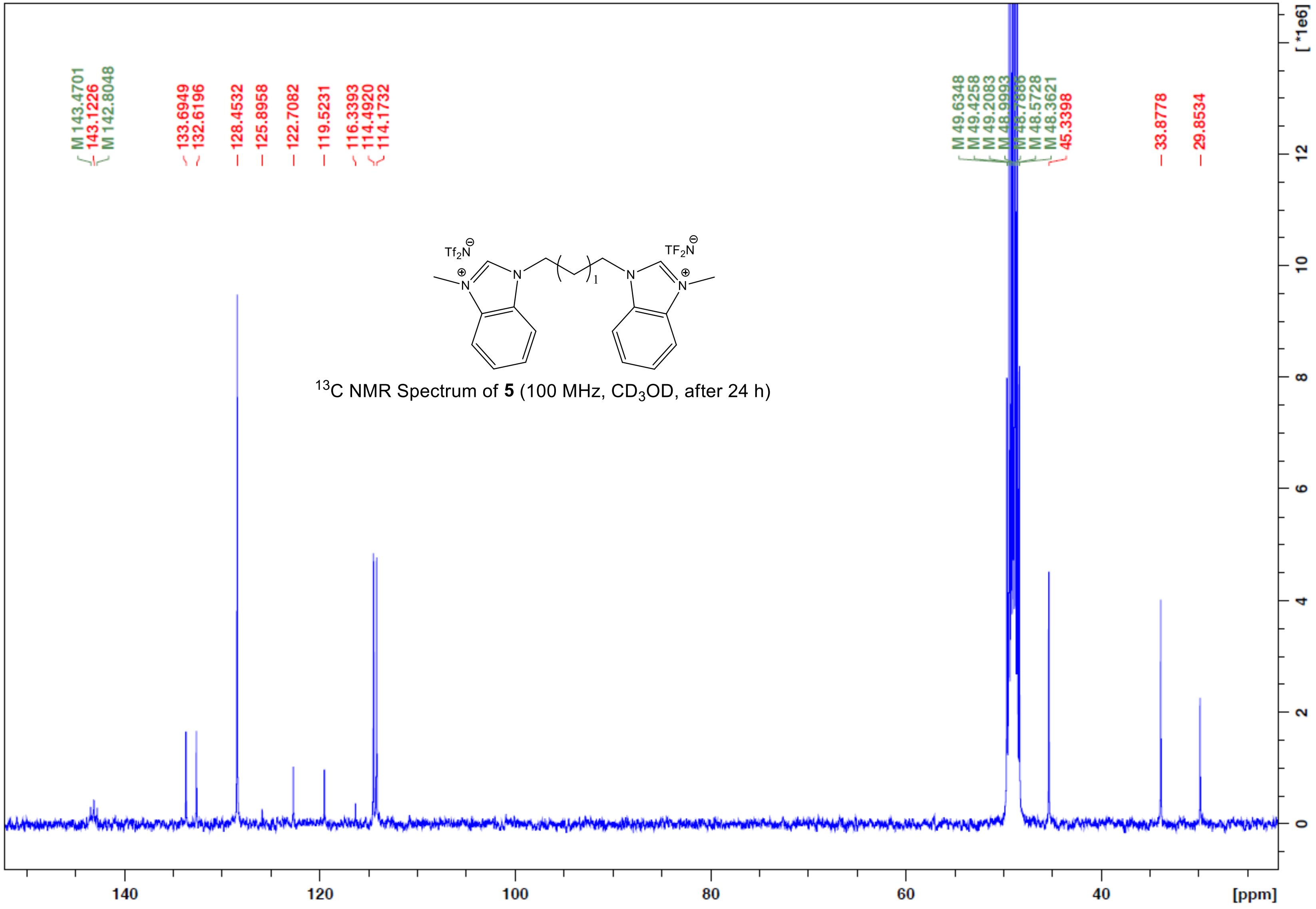

^{13}C NMR Spectrum of **5** (100 MHz, CD_3OD, after 24 h)

Figure S16. ^{13}C NMR of compound **5**.

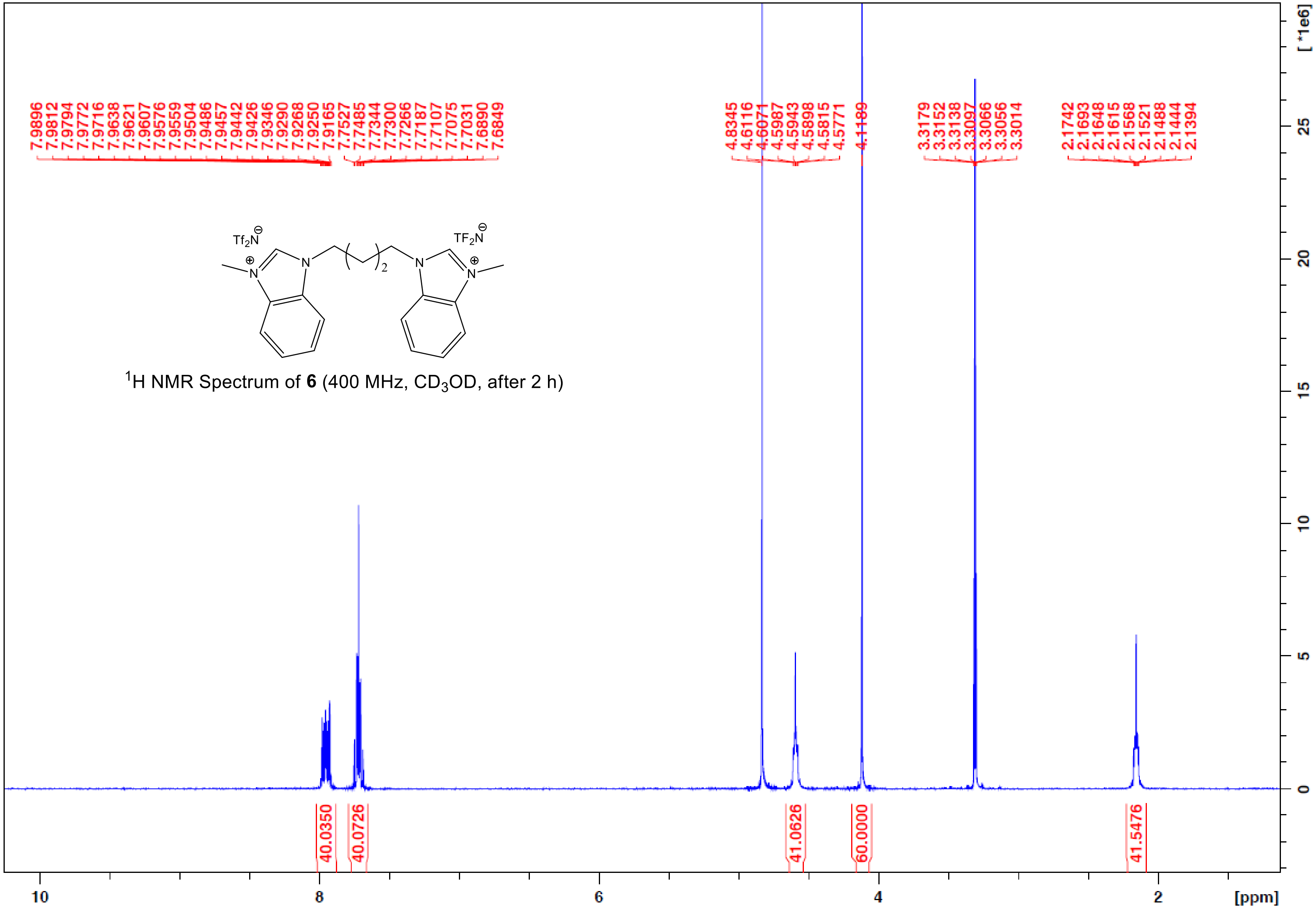

^{1}H NMR Spectrum of **6** (400 MHz, CD_3OD, after 2 h)

Figure S17. ^{1}H NMR of compound **6**.

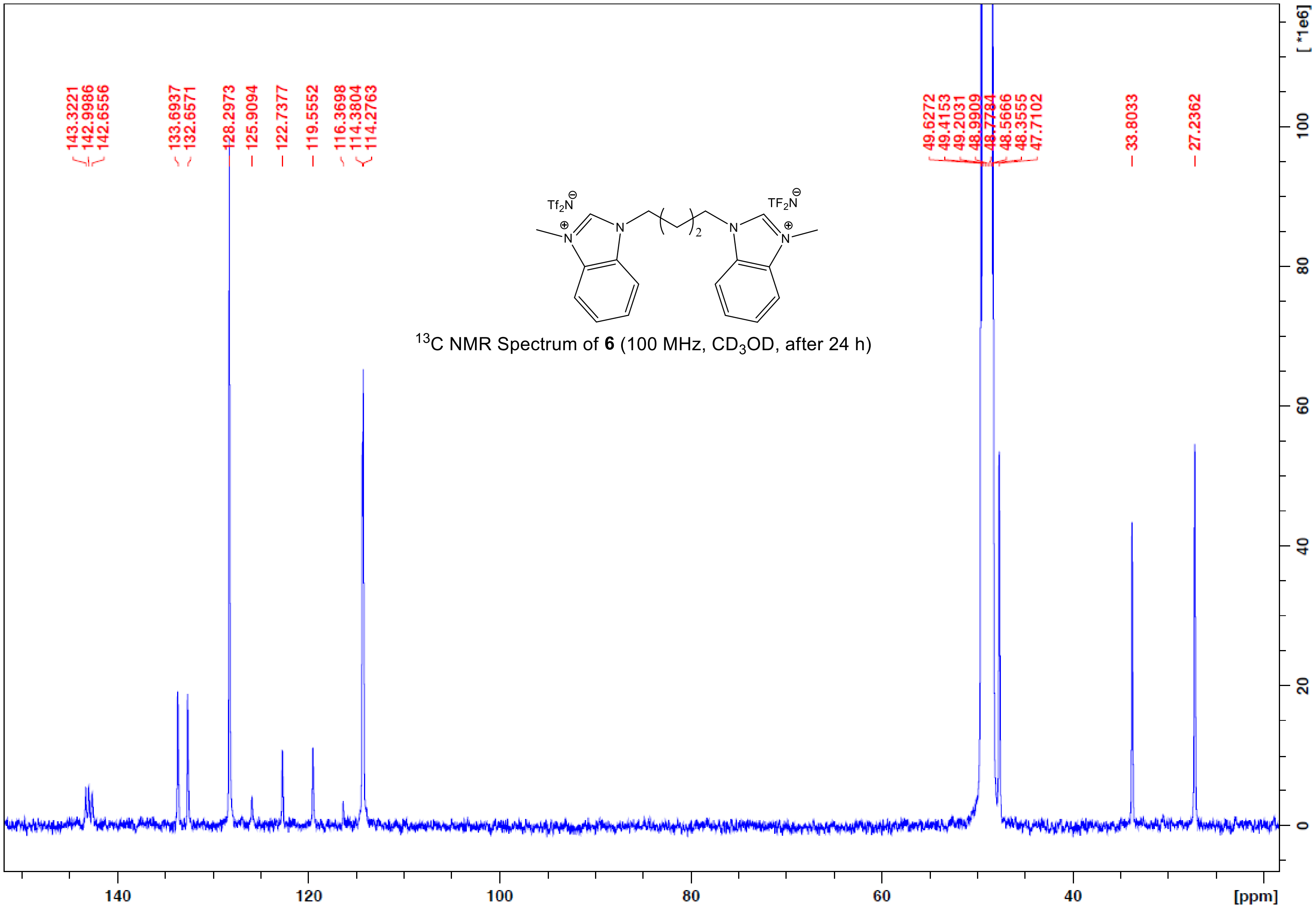

^{13}C NMR Spectrum of **6** (100 MHz, CD_3OD, after 24 h)

Figure S18. ^{13}C NMR of compound **6**.

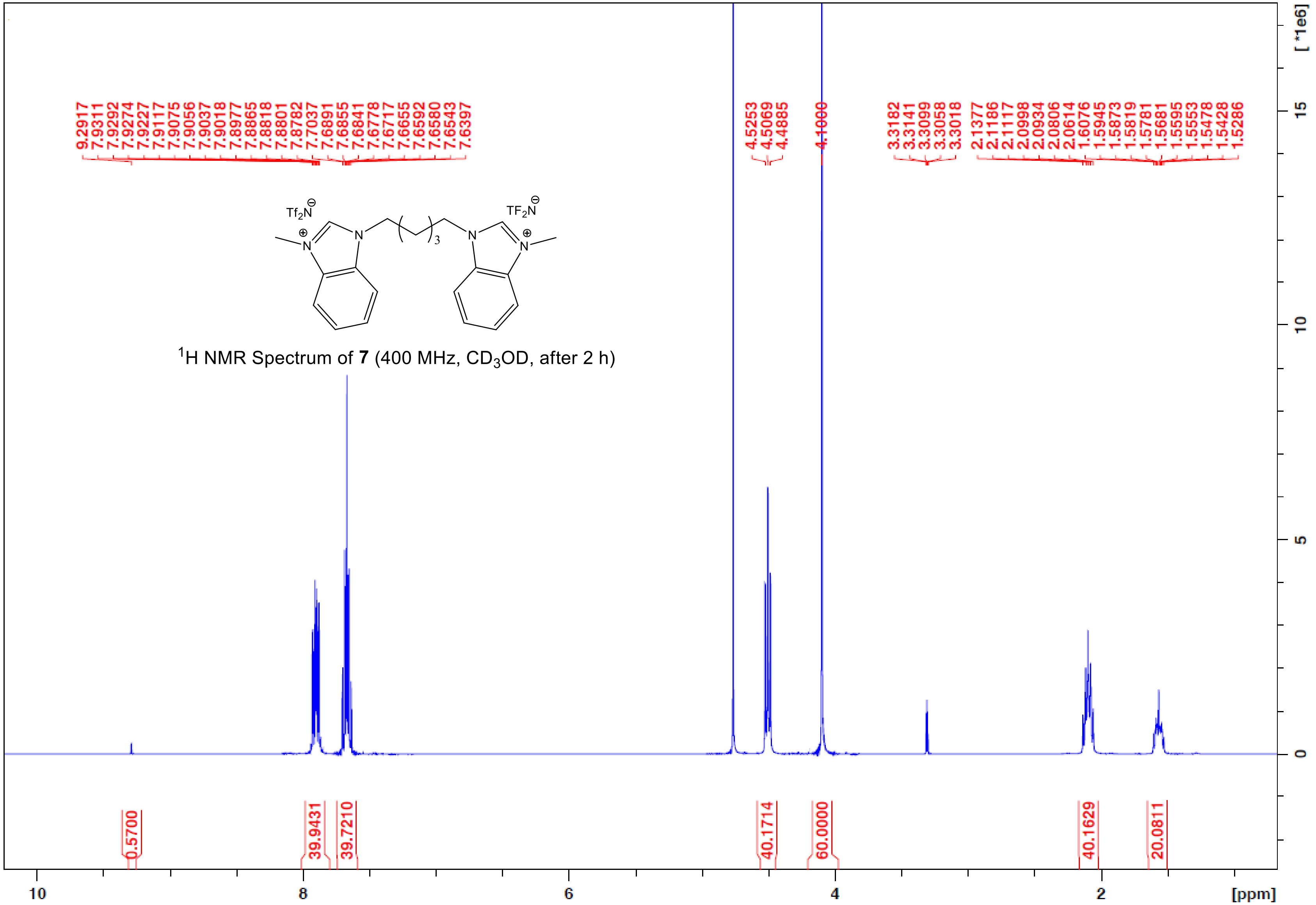

^{1}H NMR Spectrum of **7** (400 MHz, CD_3OD, after 2 h)

Figure S19. ^{1}H NMR of compound **7**.

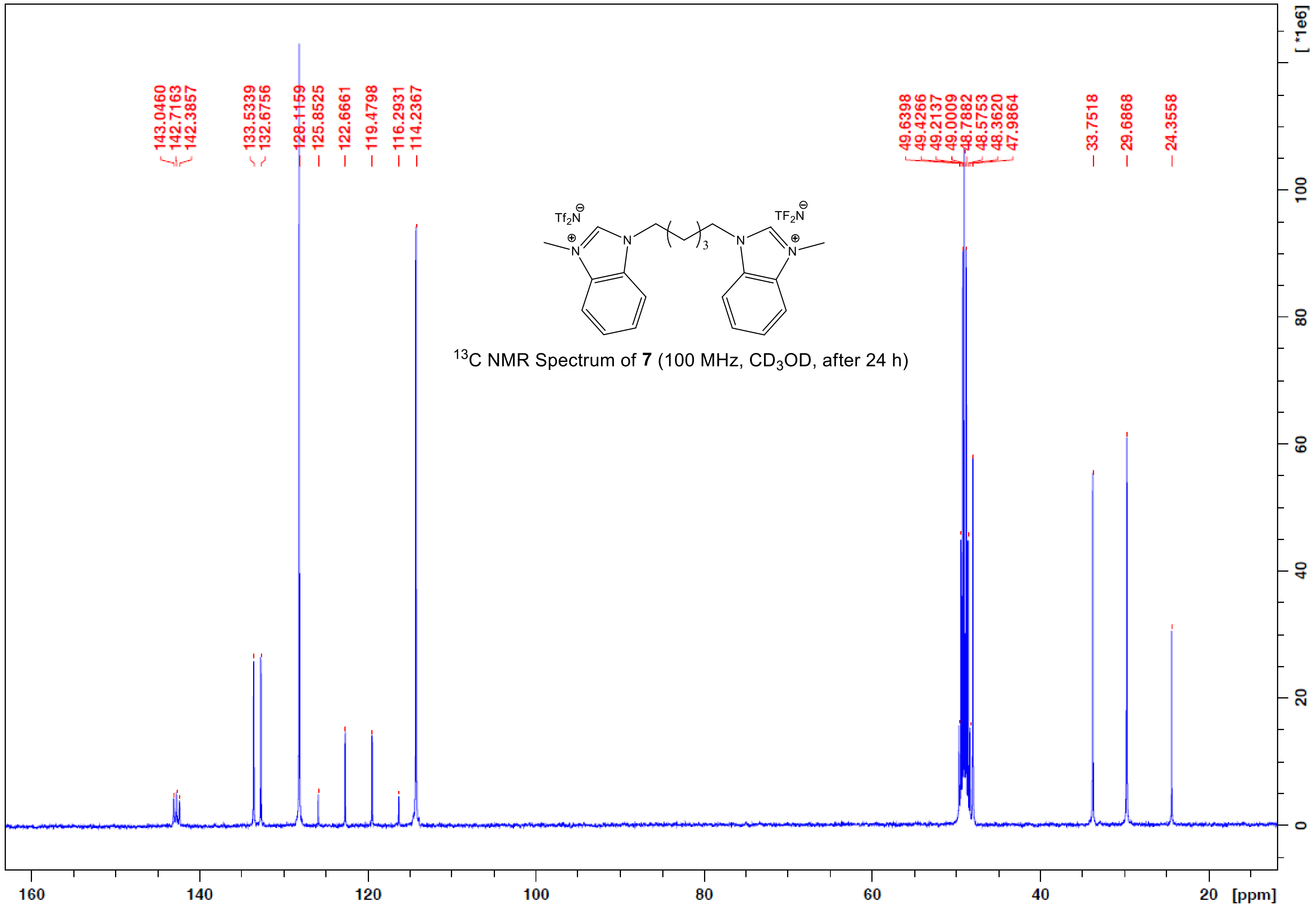

^{13}C NMR Spectrum of **7** (100 MHz, CD_3OD, after 24 h)

Figure S20. ^{13}C NMR of compound **7**.

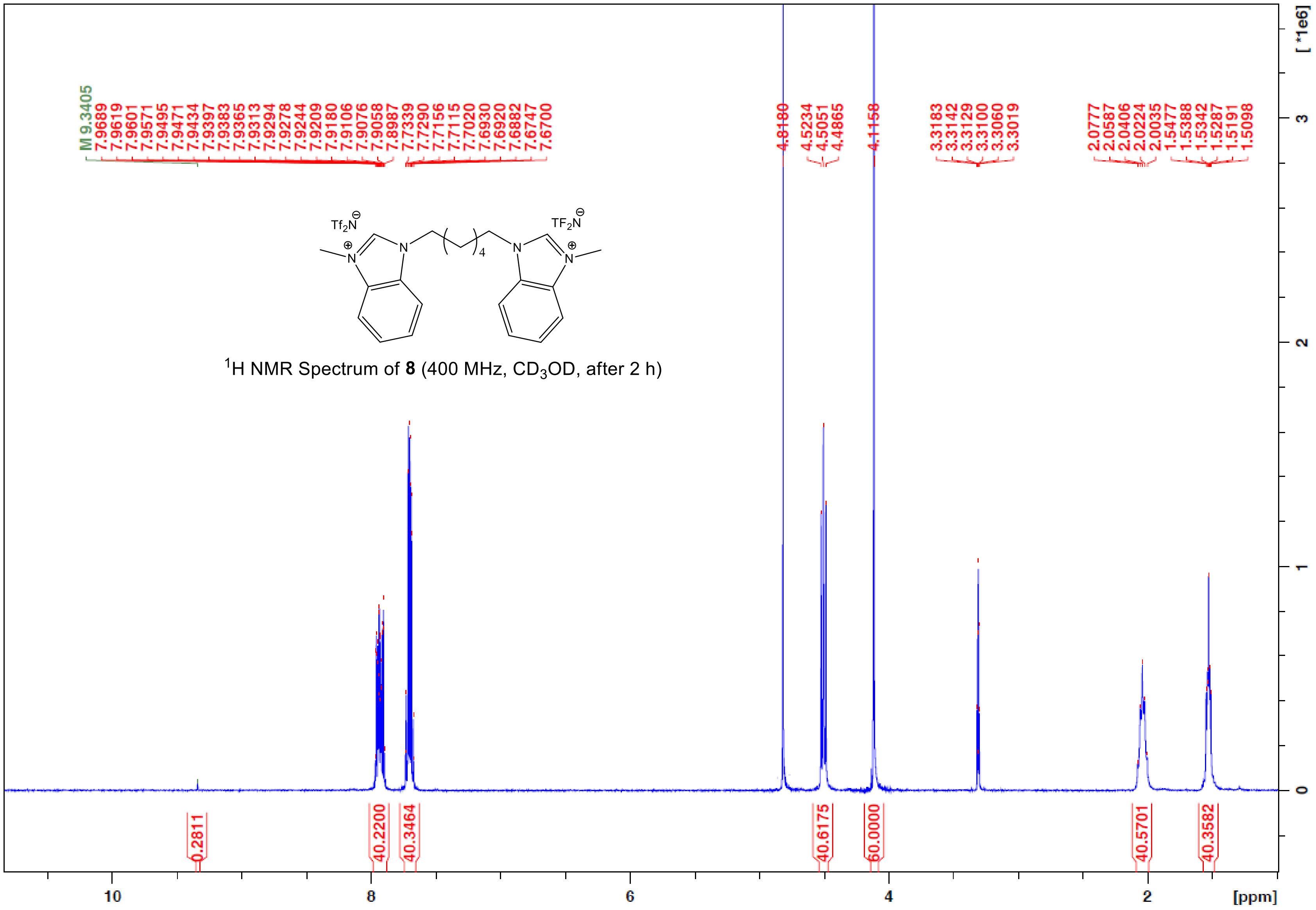

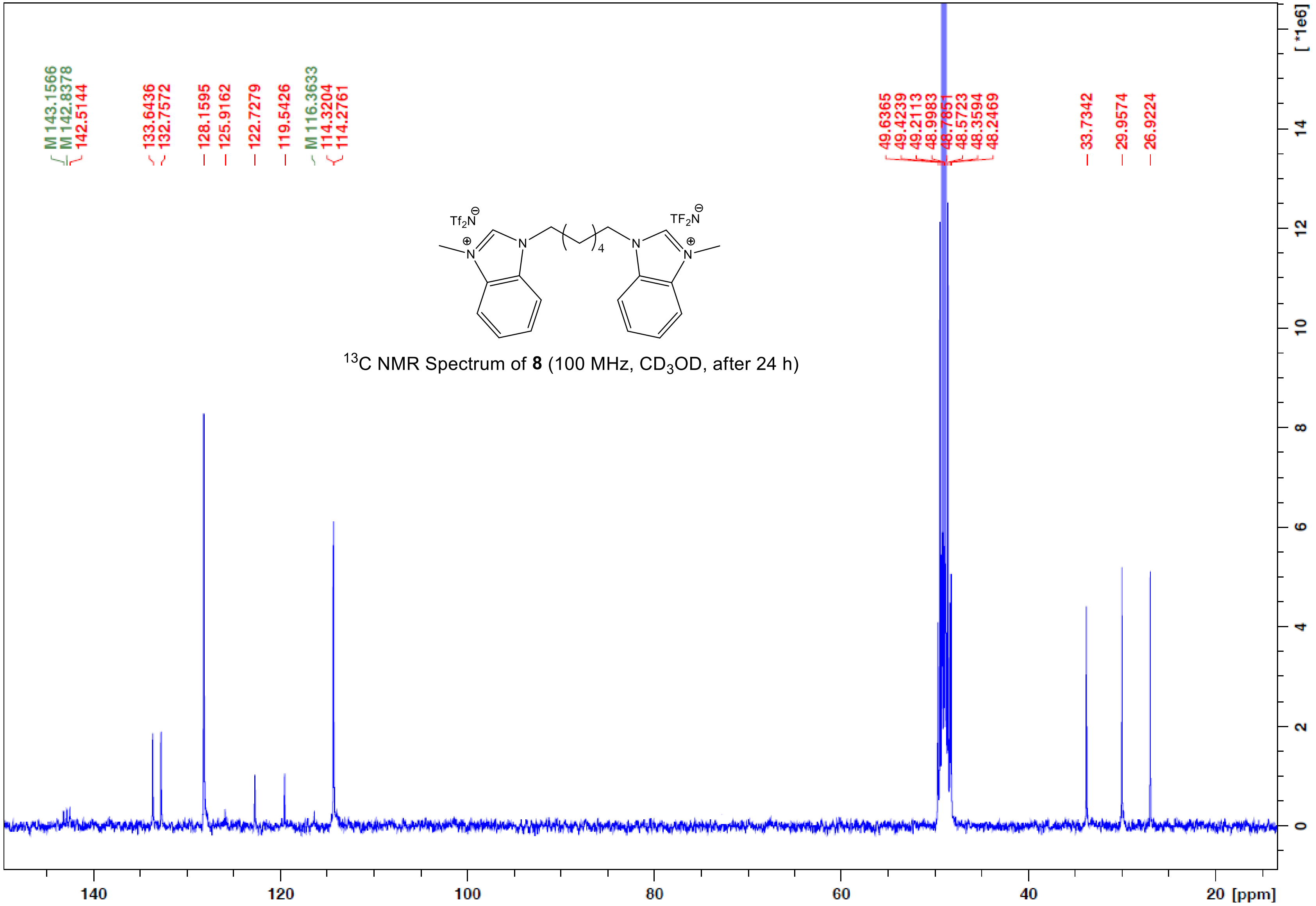
M 143.1566
M 142.8378
142.5144
133.6436
132.7572
128.1595
125.9162
122.7279
119.5426
M 116.3633
114.3204
114.2761
49.6365
49.4239
49.2113
48.9983
48.7851
48.5723
48.3594
48.2469
33.7342
29.9574
26.9224
Tf2N
TF2N
N
N
N
N
4
^{13}C NMR Spectrum of 8 (100 MHz, CD_3OD, after 24 h)
140
120
100
80
60
40
20 [ppm]
[*1e6]
14
12
10
8
6
4
2
0

Figure S22. ^{13}C NMR of compound **8**.

Thermal gravimetric analysis (TGA)

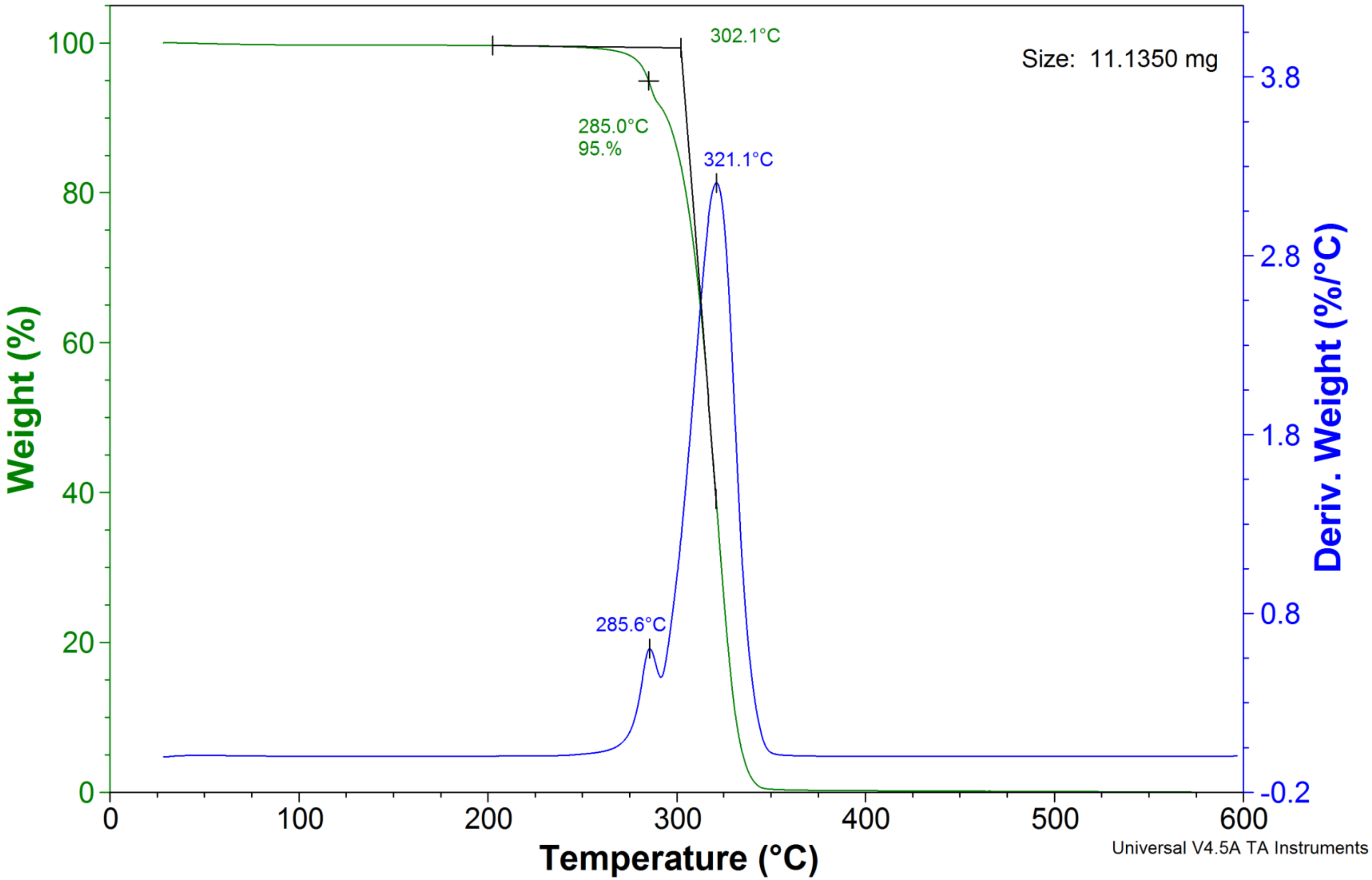

Figure S24. TGA of compound **1**.

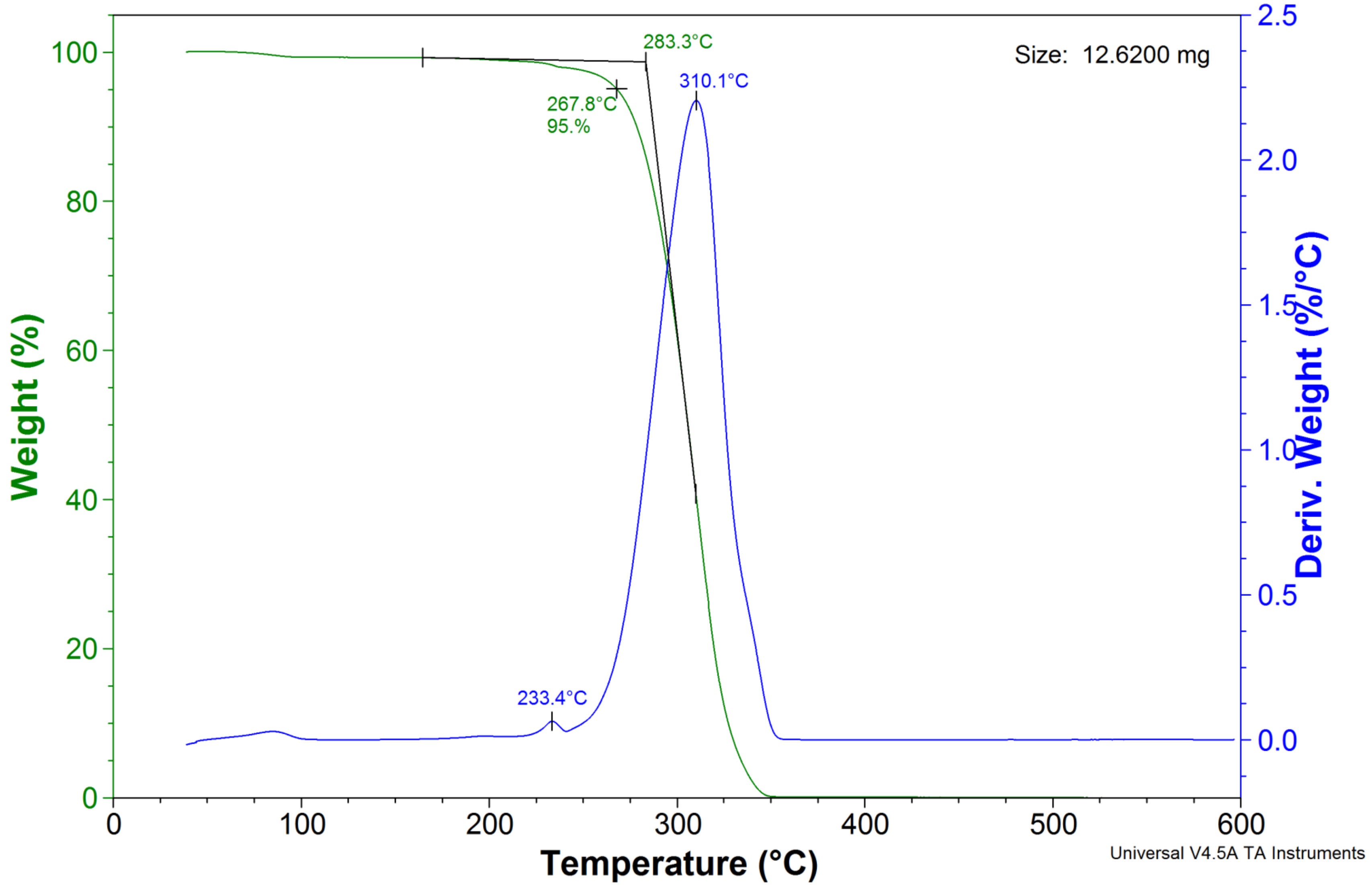

Figure S24. TGA of compound **2**.

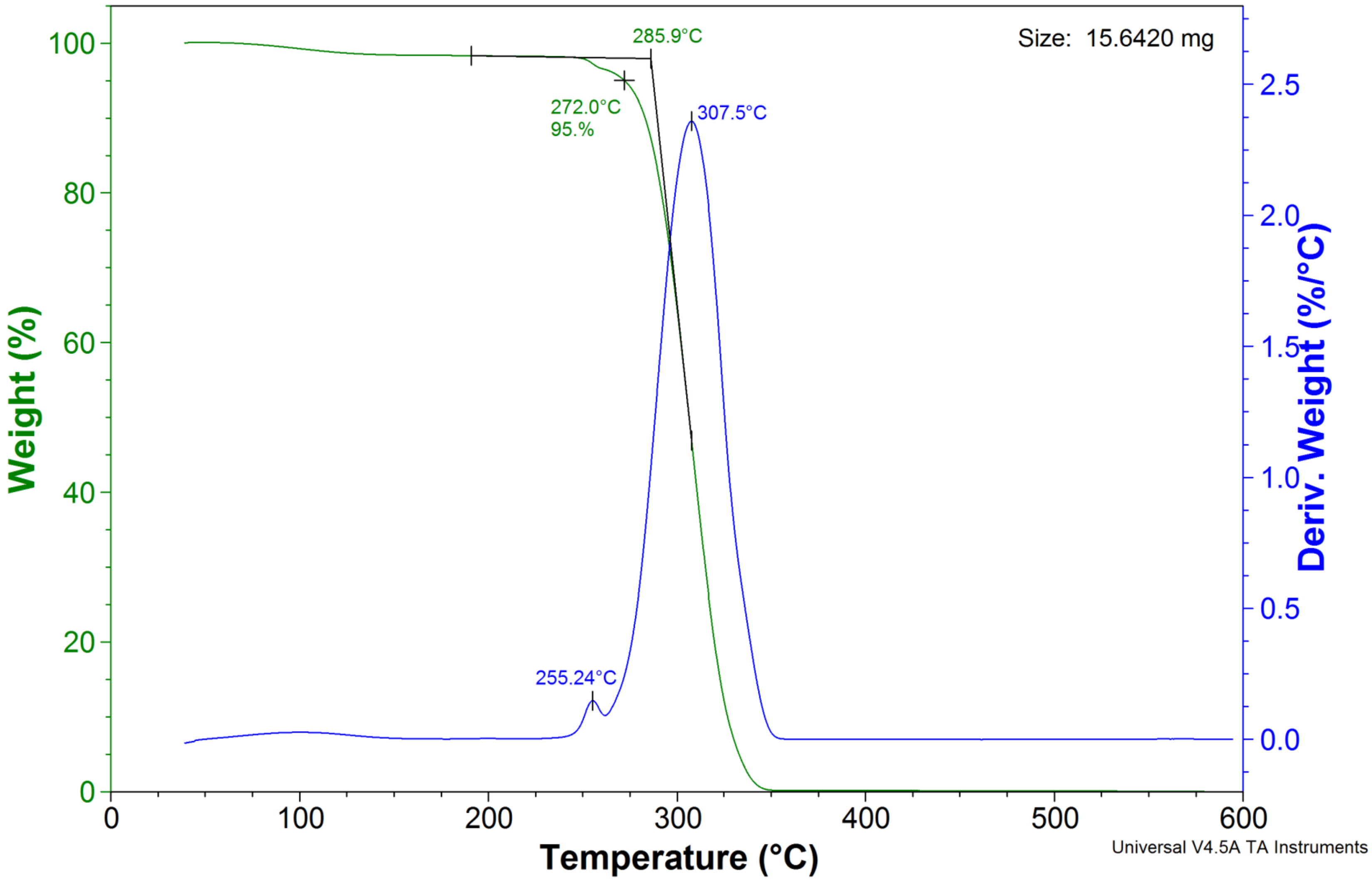

Figure S25. TGA of compound **3**.

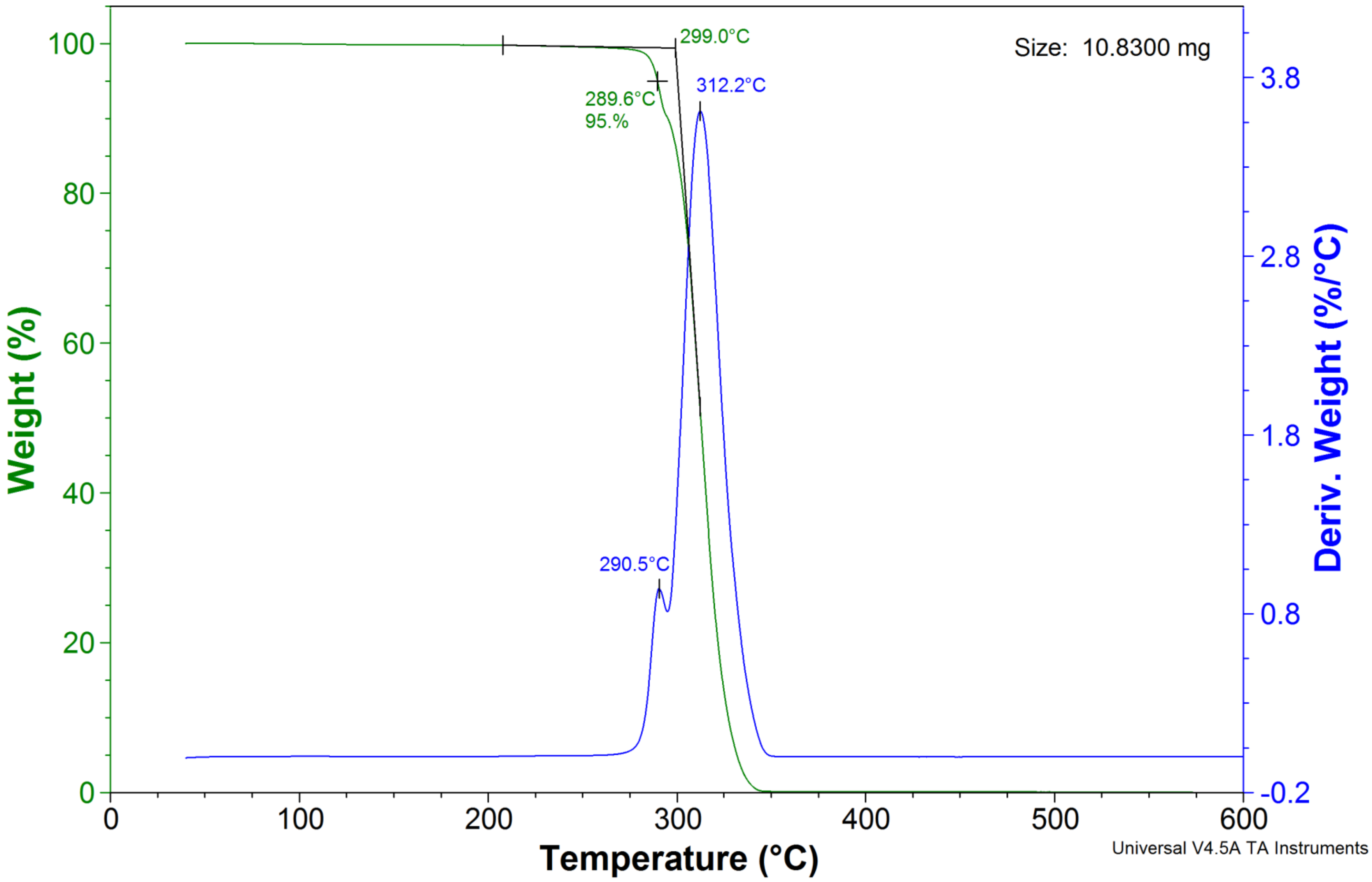

Figure S26. TGA of compound **4**.

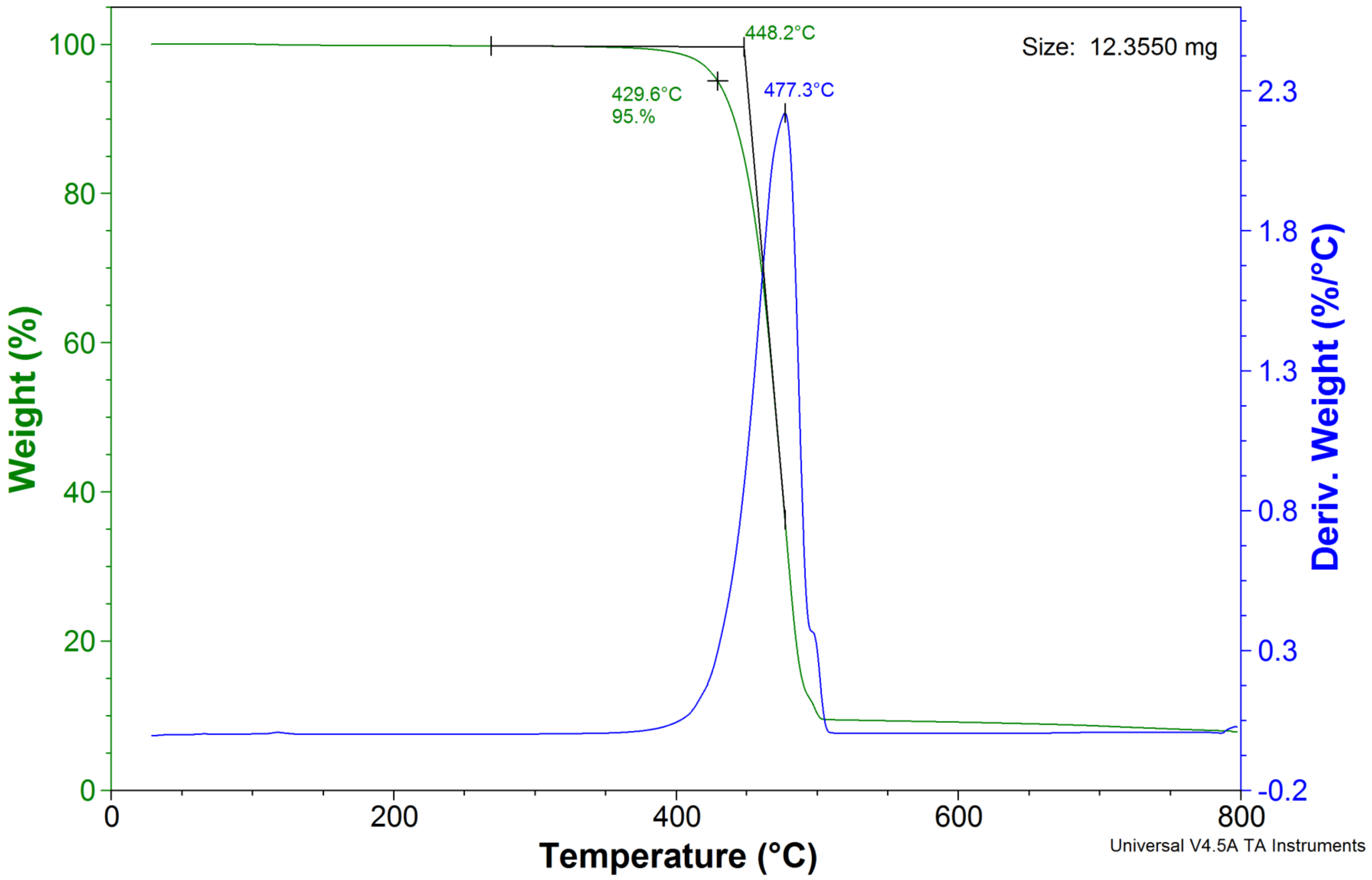

448.2°C
Size: 12.3550 mg
429.6°C
95.%
477.3°C
Weight (%)
Deriv. Weight (%/°C)
Temperature (°C)
Universal V4.5A TA Instruments

Figure S27. TGA of compound **5**.

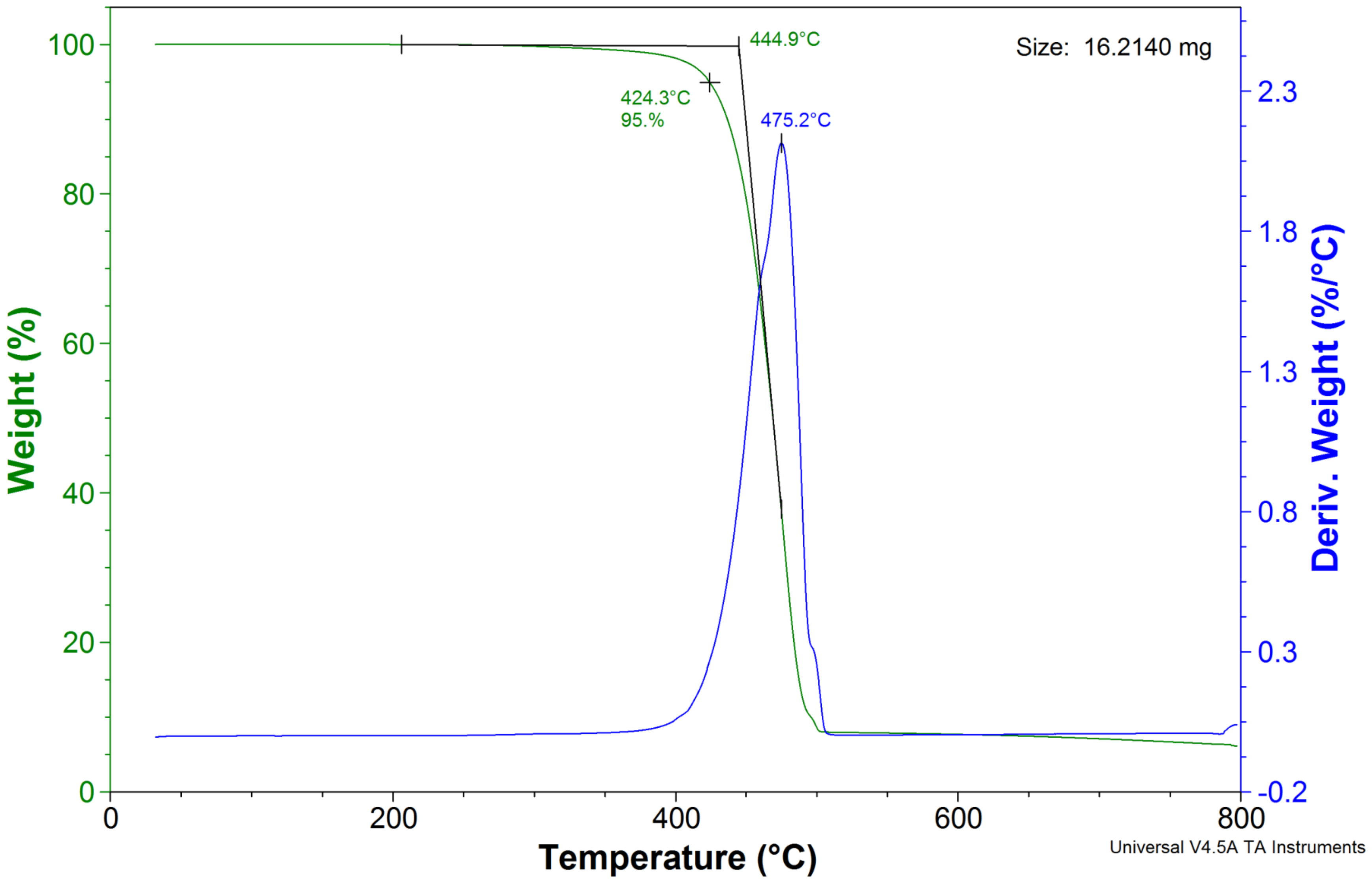

Figure S28: TGA of compound **6**.

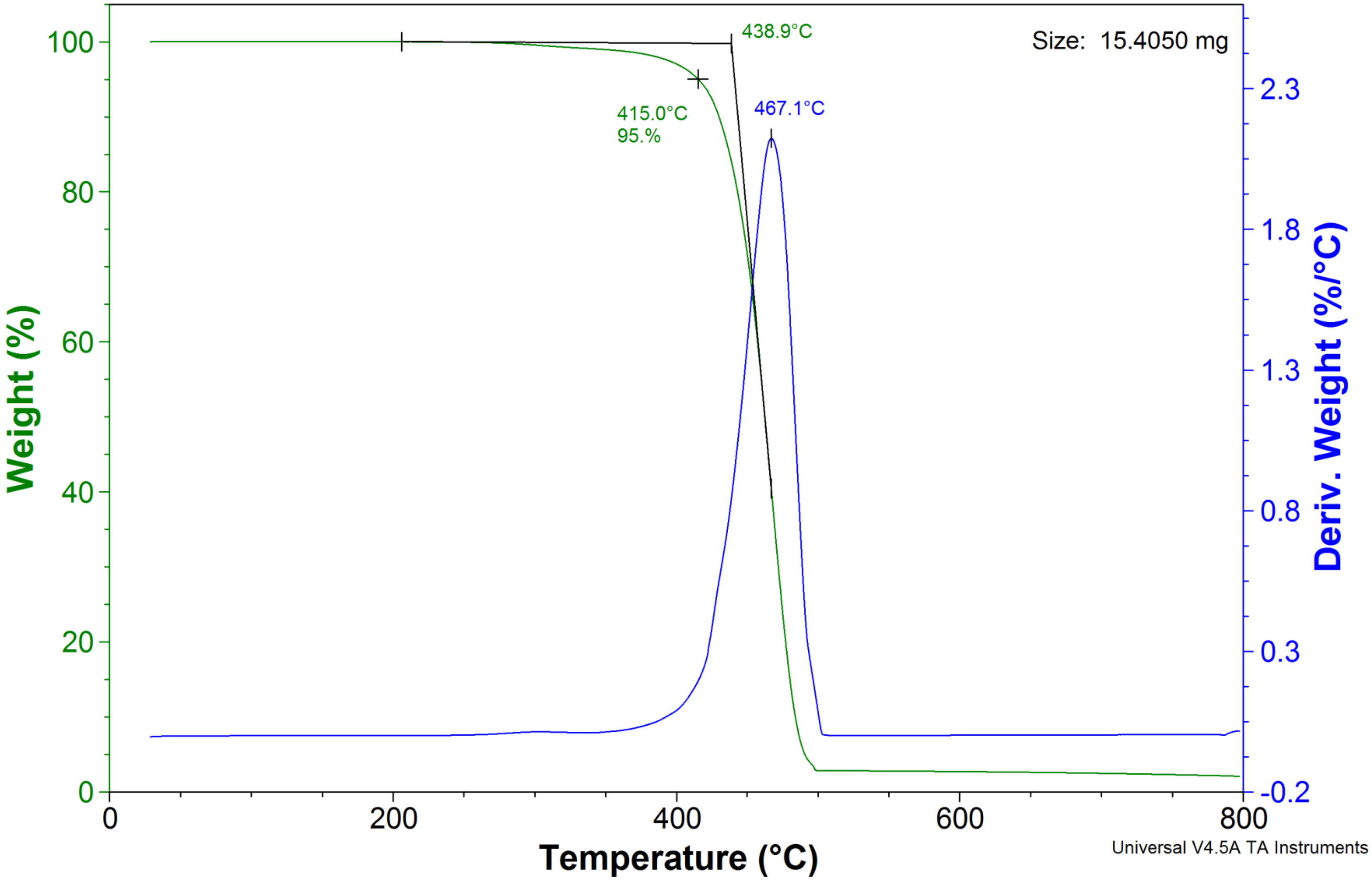

Figure S29. TGA of compound **7**.

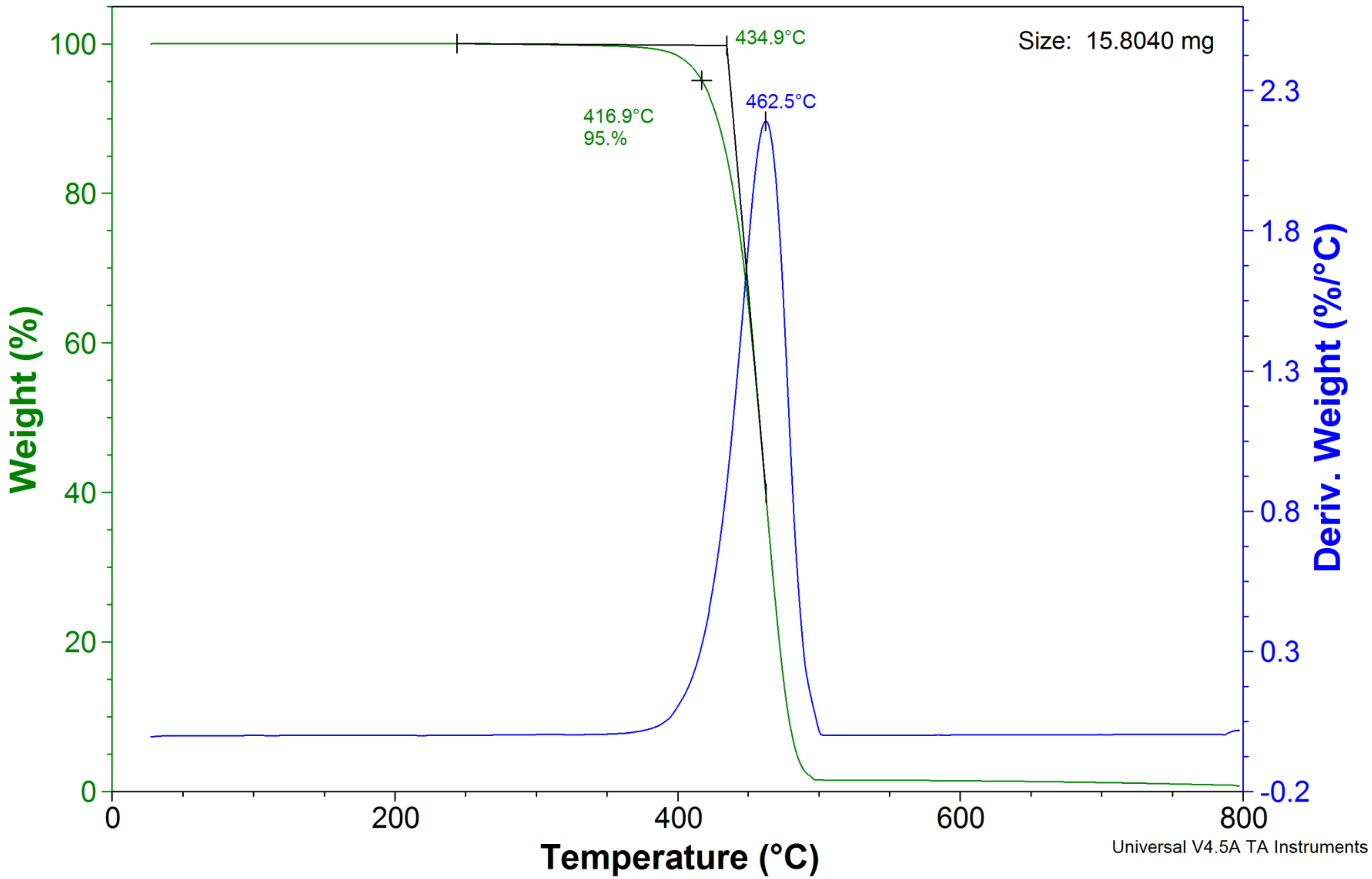

Figure S30. TGA of compound **8**.

Table S1. T_{start}, T_{onset}, and T_{peak} of the investigated ionic liquids measured at heating rate of 10 °C/min.

**Br^- anion**				
**ILs**	**n**	**$T_{start\ (5\%)}$ (°C)**	**T_{onset} (°C)**	**T_{peak} (°C)**
$[C_3(C_1BenzIm)_2]Br_2$	***1***	285.0	302.1	285.6<br>321.1
$[C_4(C_1BenzIm)_2]Br_2$	***2***	267.8	283.3	233.4<br>310.1
$[C_5(C_1BenzIm)_2]Br_2$	***3***	272.0	285.9	255.2<br>307.5
$[C_6(C_1BenzIm)_2]Br_2$	***4***	289.6	299.0	290.5<br>312.2
**$[Tf_2N]^-$ anion**				
$[C_3(C_1BenzIm)_2][Tf_2N]_2$	***5***	429.6	448.2	477.2
$[C_4(C_1BenzIm)_2][Tf_2N]_2$	***6***	424.3	444.9	475.2
$[C_5(C_1BenzIm)_2][Tf_2N]_2$	***7***	415.0	438.9	467.1
$[C_6(C_1BenzIm)_2][Tf_2N]_2$	***8***	416.9	434.9	462.5

Differential scanning calorimetry (DSC)

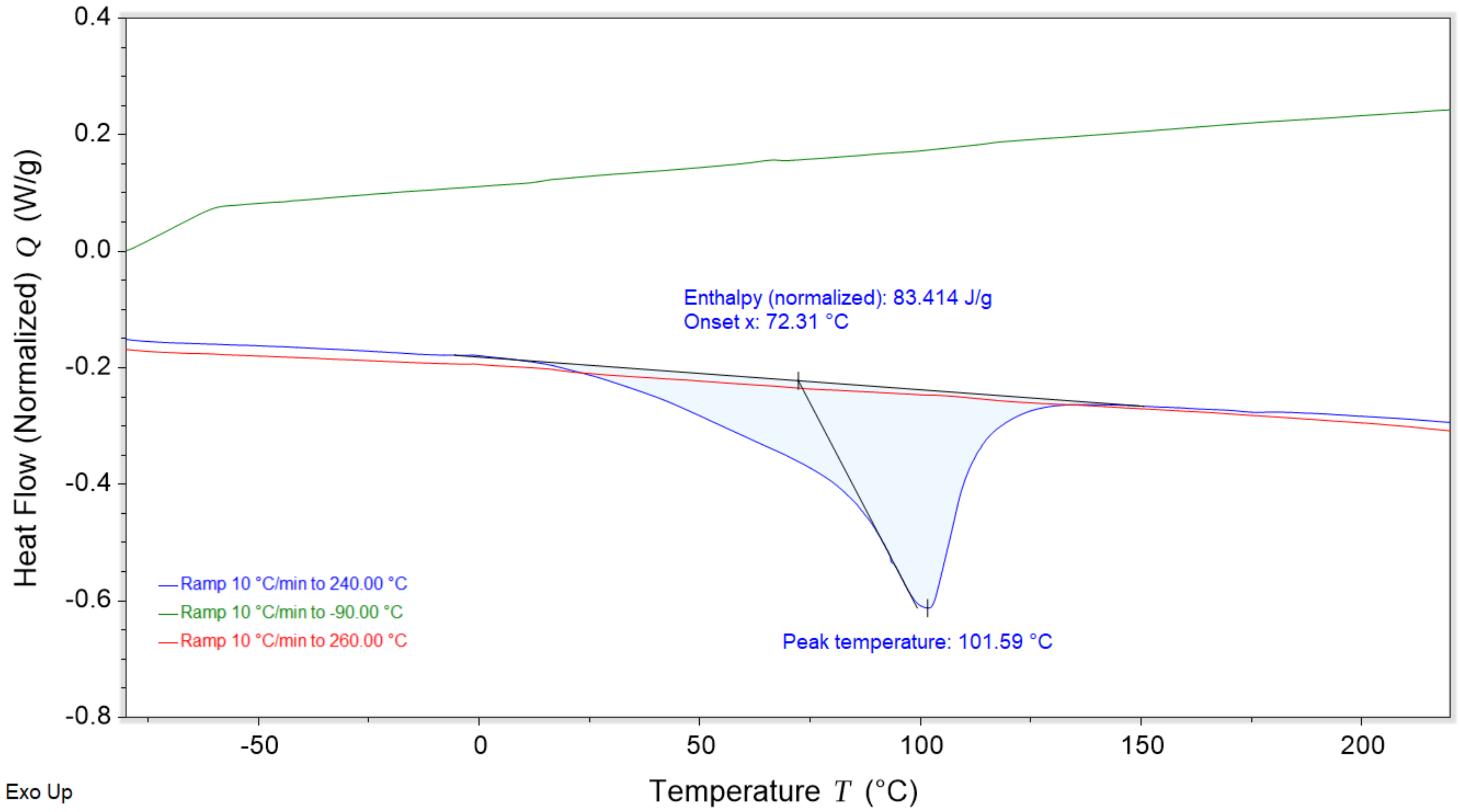

Figure S31. DSC of compound **1**.

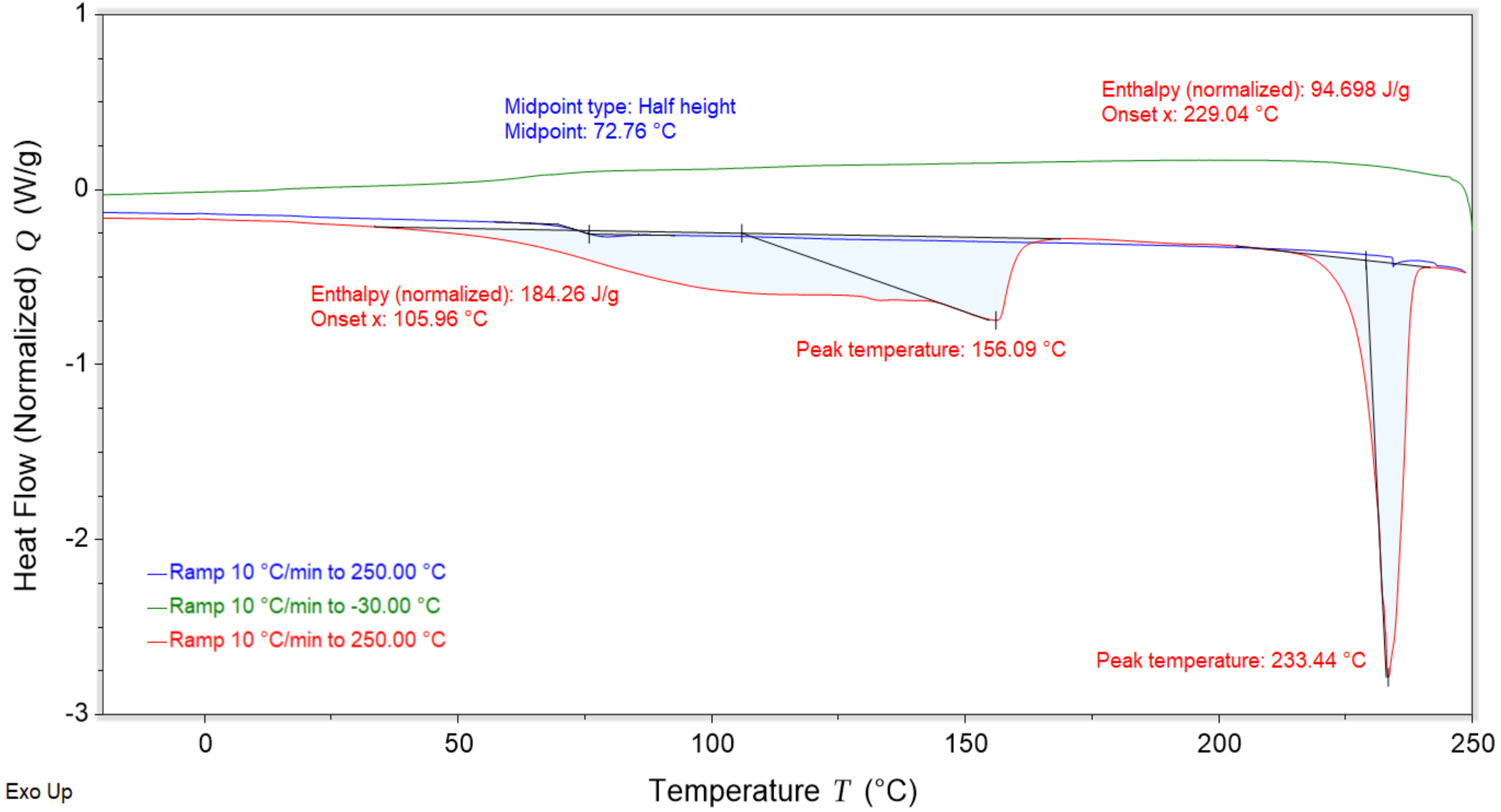

Figure S32. DSC of compound **2**.

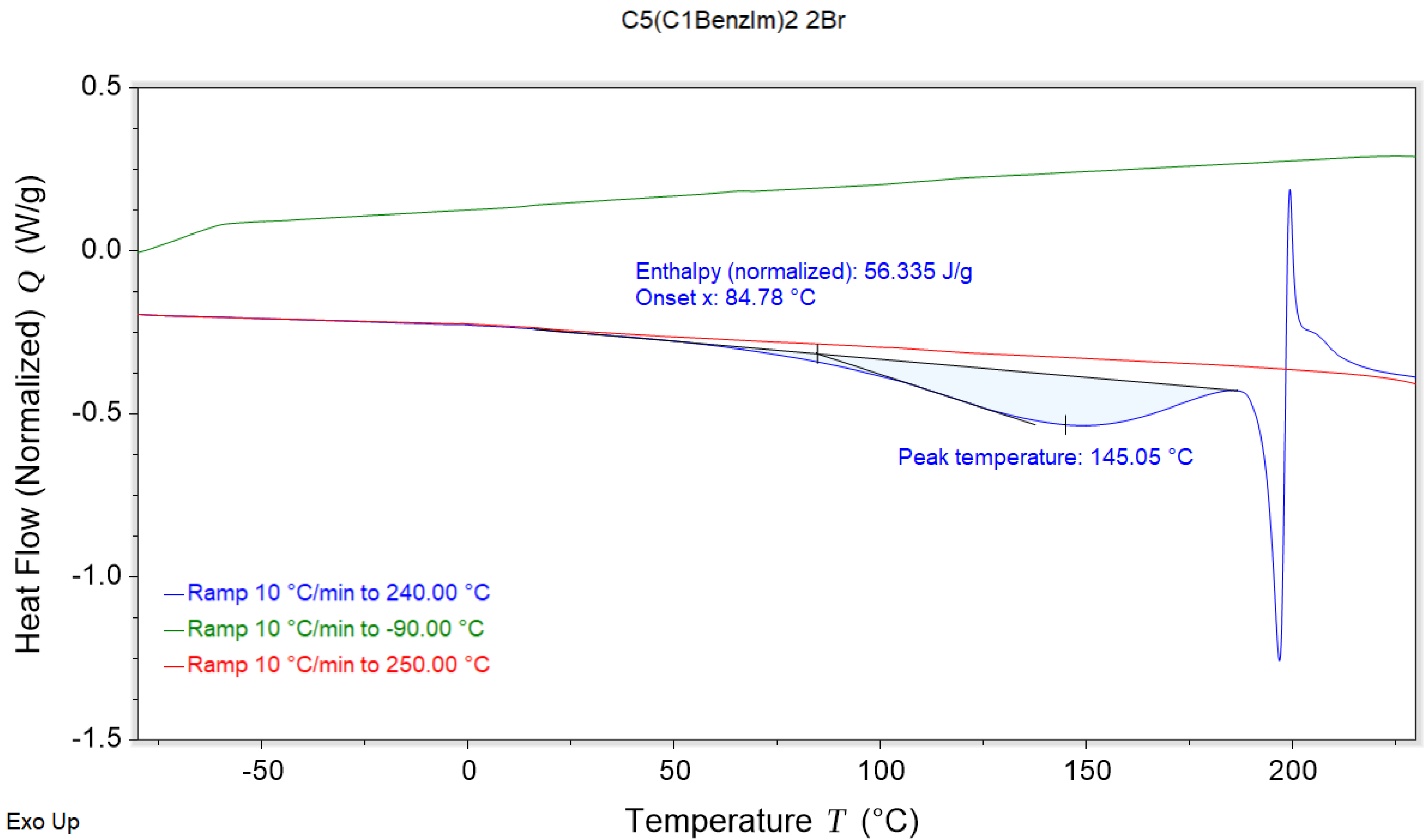

Figure S33. DSC of compound **3**.

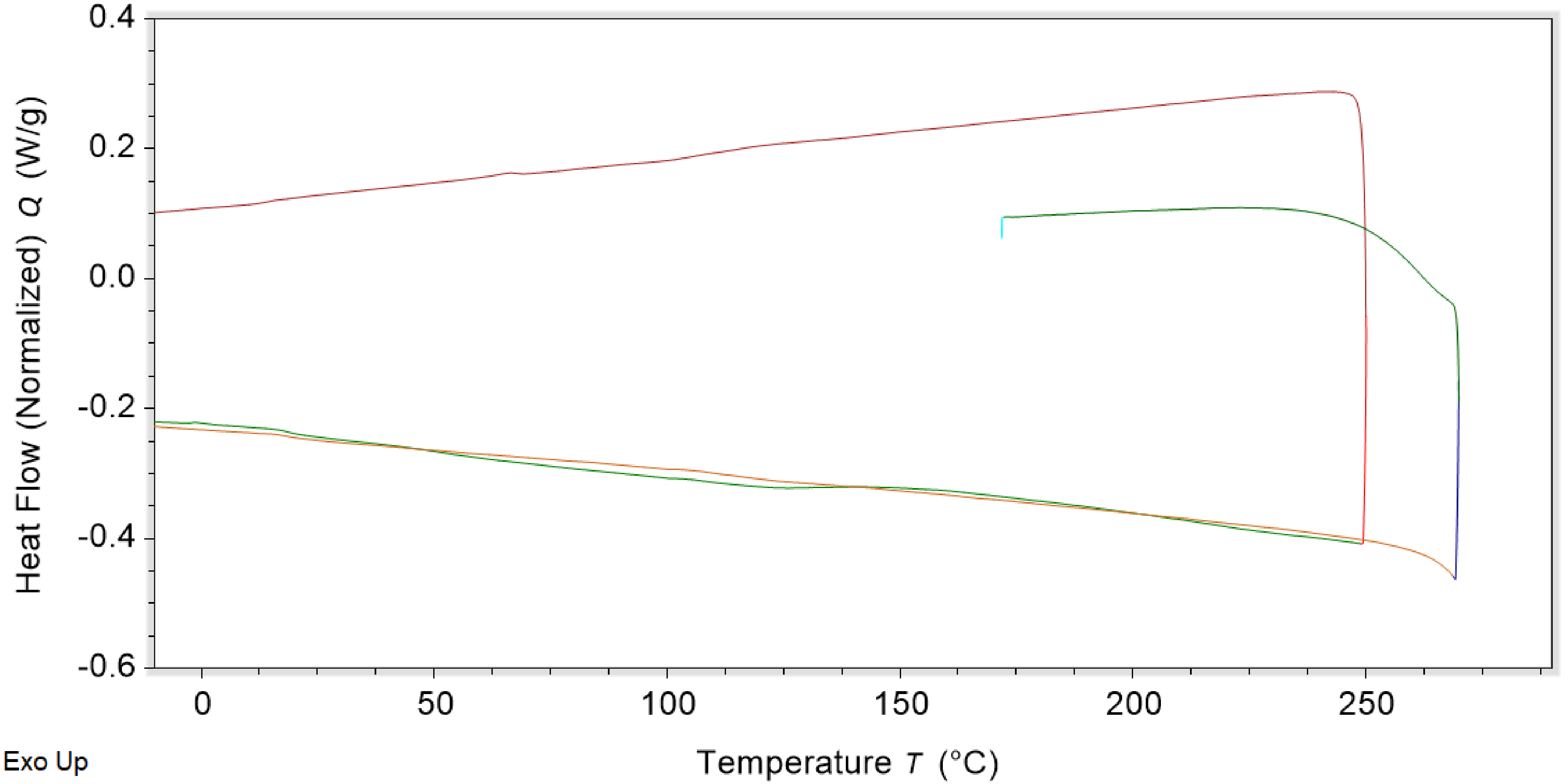

Figure S34. DSC of compound **4**.

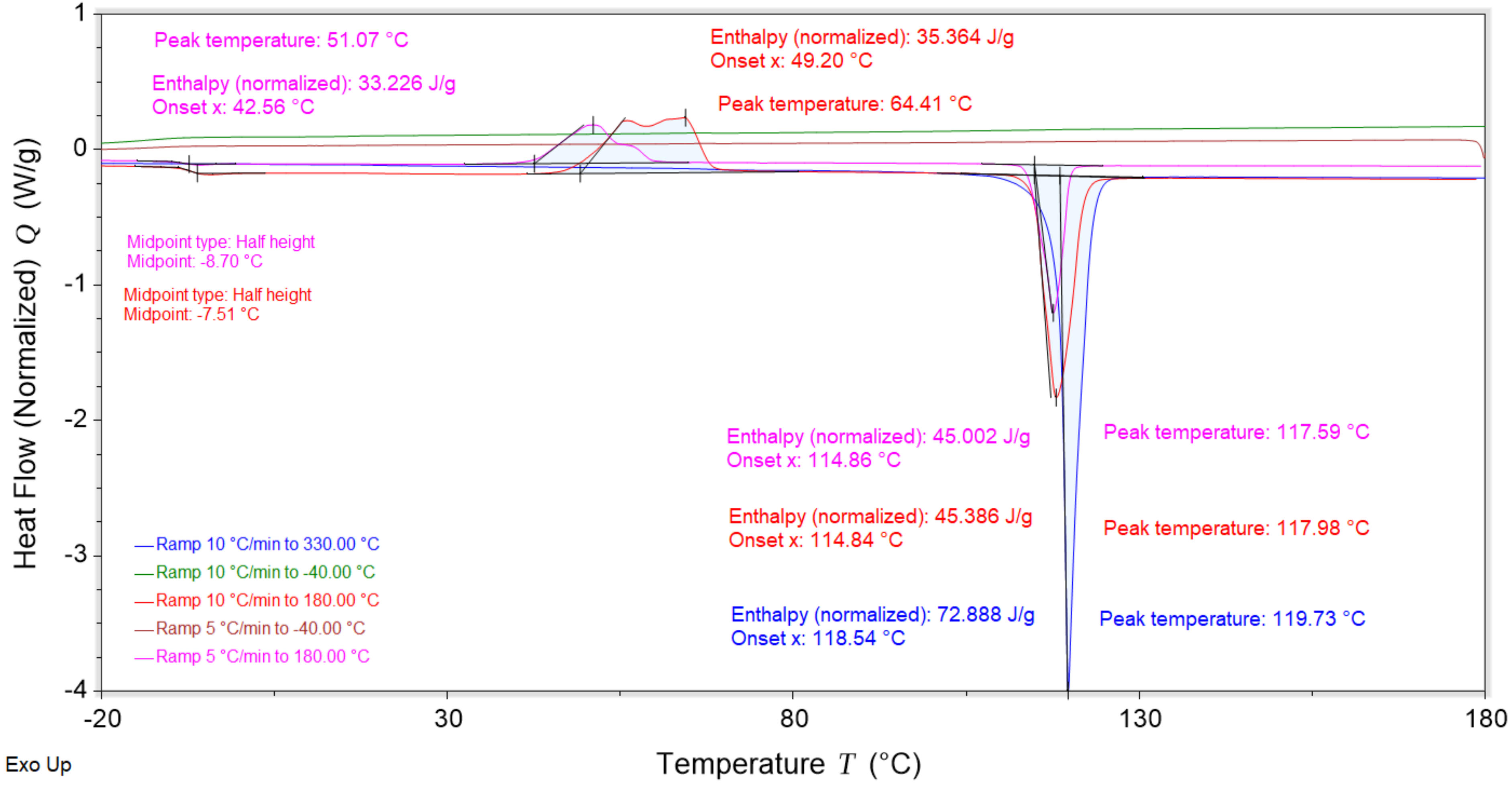

Figure S35. DSC of compound **5**.

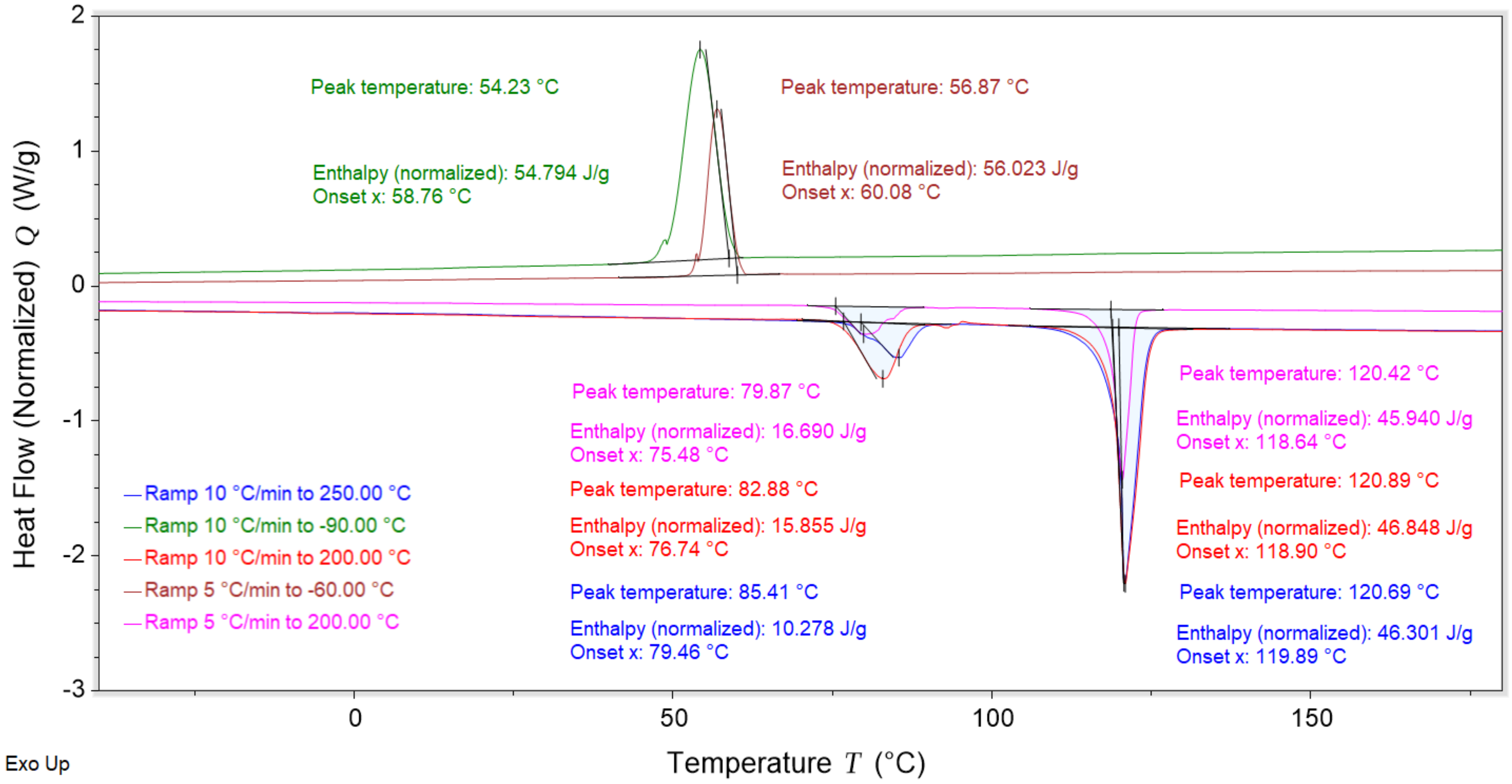

Figure S36. DSC of compound **6**.

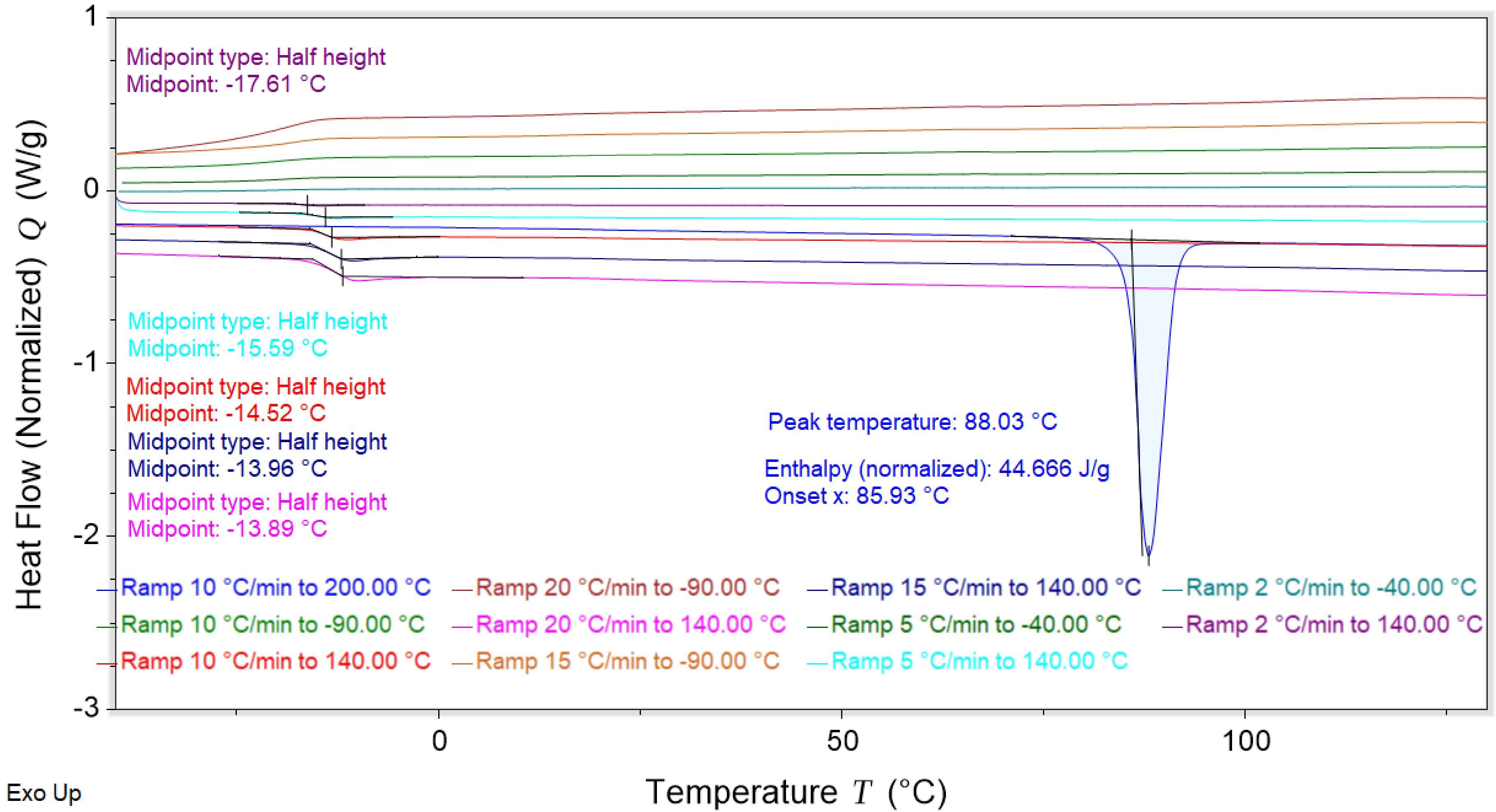

Figure S37. DSC of compound **7**.

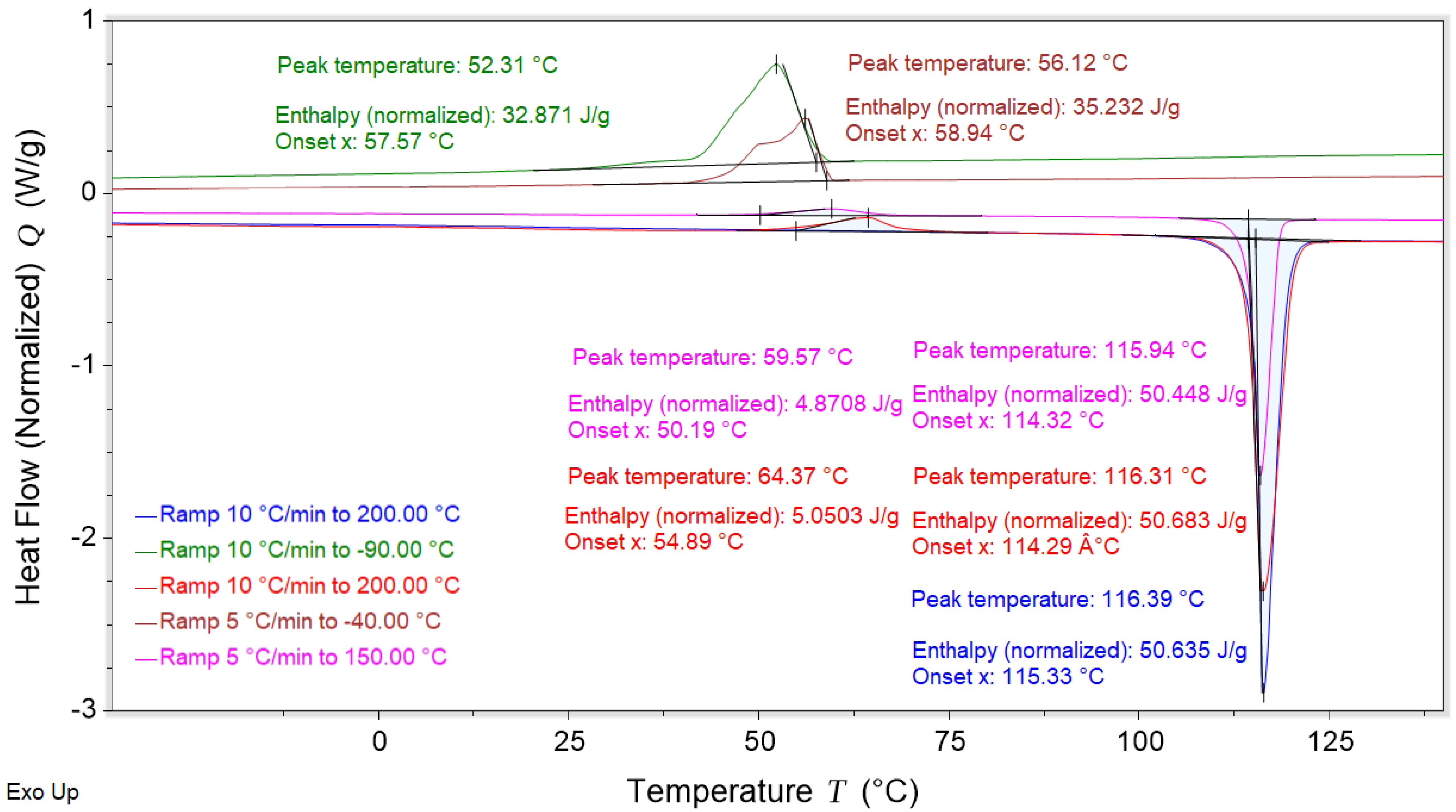

Figure S38. DSC of compound **8**.

Table S2. Melting temperature (T_m), and enthalpy (H_m) of the investigated ionic liquids measured at first heating run.

ILs	n	T_m (°C)	H_m (KJ/mol)
$[C_3(C_1BenzIm)_2]Br_2$	*1*	72.3[a]	38.89[a]
$[C_4(C_1BenzIm)_2]Br_2$	*2*	106.0[a]	45.48[a]
$[C_5(C_1BenzIm)_2]Br_2$	*3*	84.8[a]	27.85[a]
$[C_6(C_1BenzIm)_2]Br_2$	*4*	--	--
$[C_3(C_1BenzIm)_2][Tf_2N]_2$	*5*	118.5	63.17
$[C_4(C_1BenzIm)_2][Tf_2N]_2$	*6*	79.5<br>119.9	8.91<br>40.77
$[C_5(C_1BenzIm)_2][Tf_2N]_2$	*7*	85.9	39.96
$[C_6(C_1BenzIm)_2][Tf_2N]_2$	*8*	115.3	46.02

[a] melting of hydrated form.

Table S3. Glass transition temperature (T_g), crystallization temperature (T_c), cold crystallization temperature (T_{cc}),and melting temperature (T_m) of the investigated ionic liquids measured at second heating run.

ILs		Temperature (°C)							
		10 °C/min				5 °C/min			
	n	T_g	T_c	T_{cc}	T_m	T_g	T_c	T_{cc}	T_m
$[C_3(C_1BenzIm)_2][Tf_2N]_2$	*5*	-7.5	--	49.2	114.8	-8.7	--	42.6	114.7
$[C_4(C_1BenzIm)_2][Tf_2N]_2$	*6*	--	58.8	--	76.7<br>118.9	--	60.1	--	75.5<br>118.6
$[C_5(C_1BenzIm)_2][Tf_2N]_2$	*7*	-14.5	--	--	--	-15.6	--	--	--
$[C_6(C_1BenzIm)_2][Tf_2N]_2$	*8*	--	57.6	54.9	114.3	--	58.9	50.2	114.3

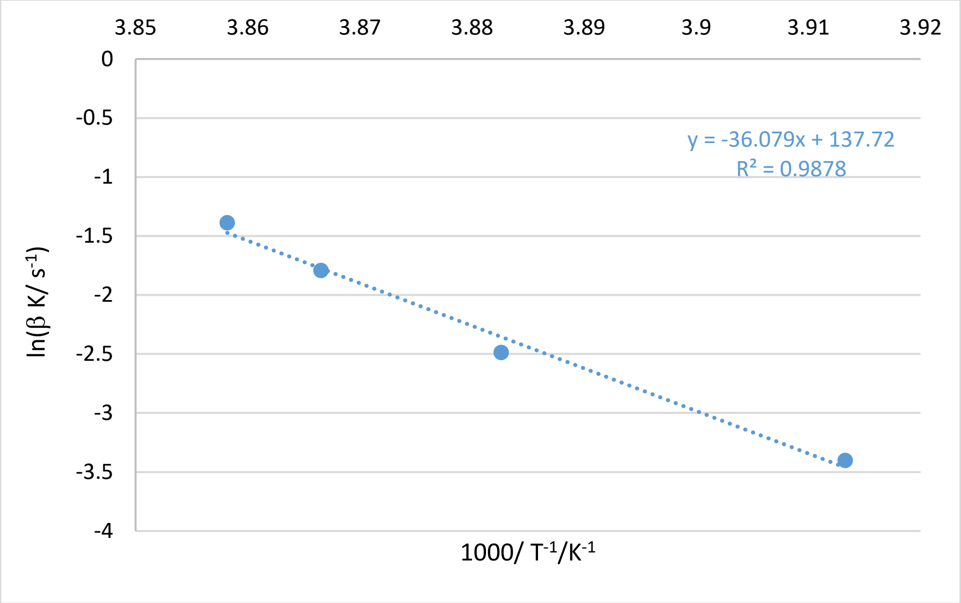

Table S4. Apparent activation energy (E_a), consequently its fragility index (m), and glass transition temperature (T_g/K) of compounds **7**

	n	Slope	T_g (K)	E_a (KJ/mol)	m
$[C_5(C_1BenzIm)_2][Tf_2N]_2$	7	-36.079	258.63	299.98	47.9

Cyclic voltammetry

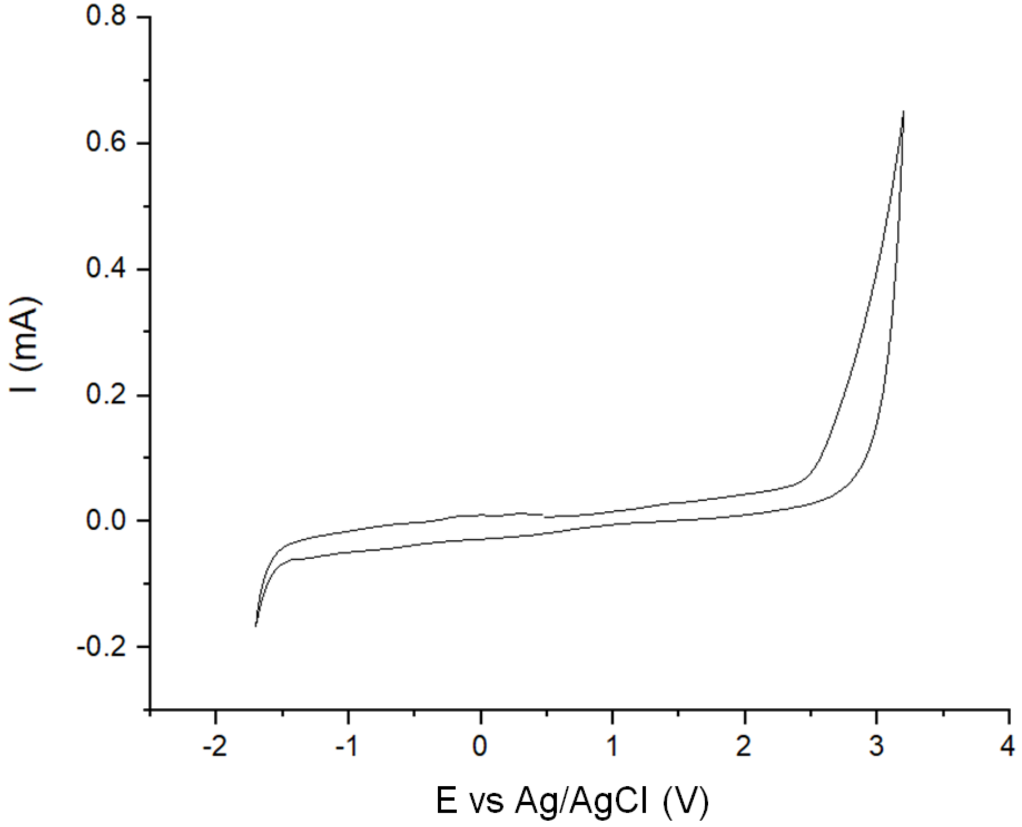

Figure S39. Cyclic voltammetry of compound **5**.

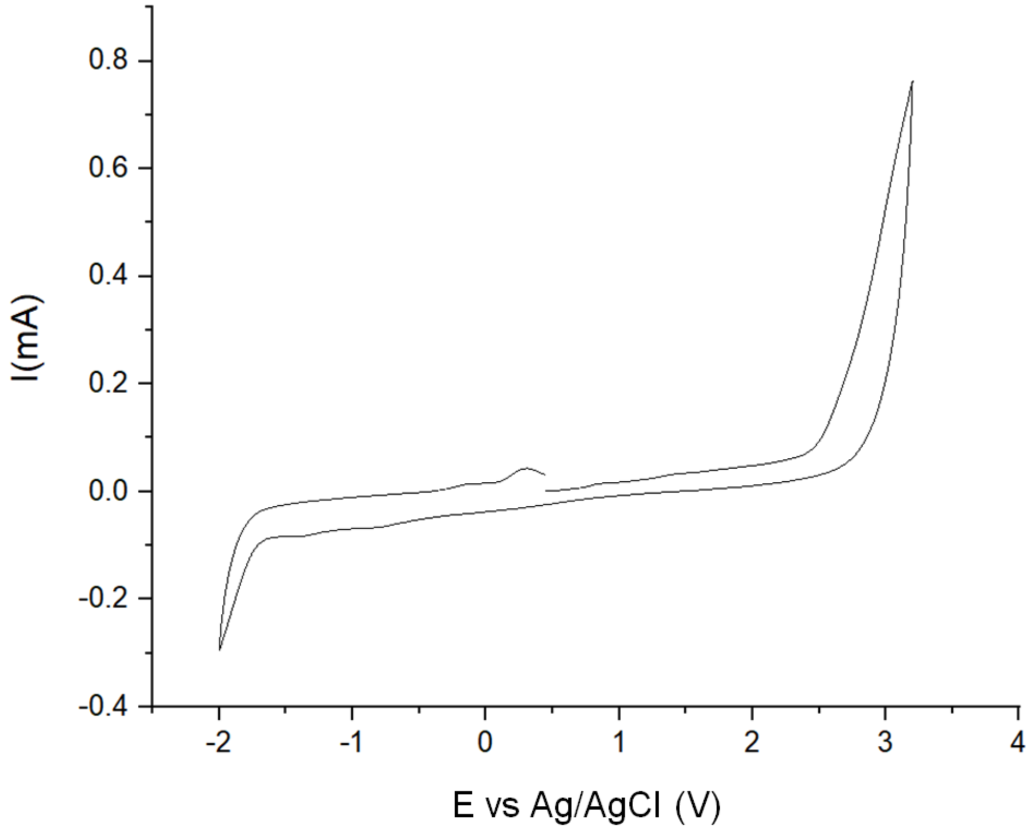

Figure S40. Cyclic voltammetry of compound **6**.

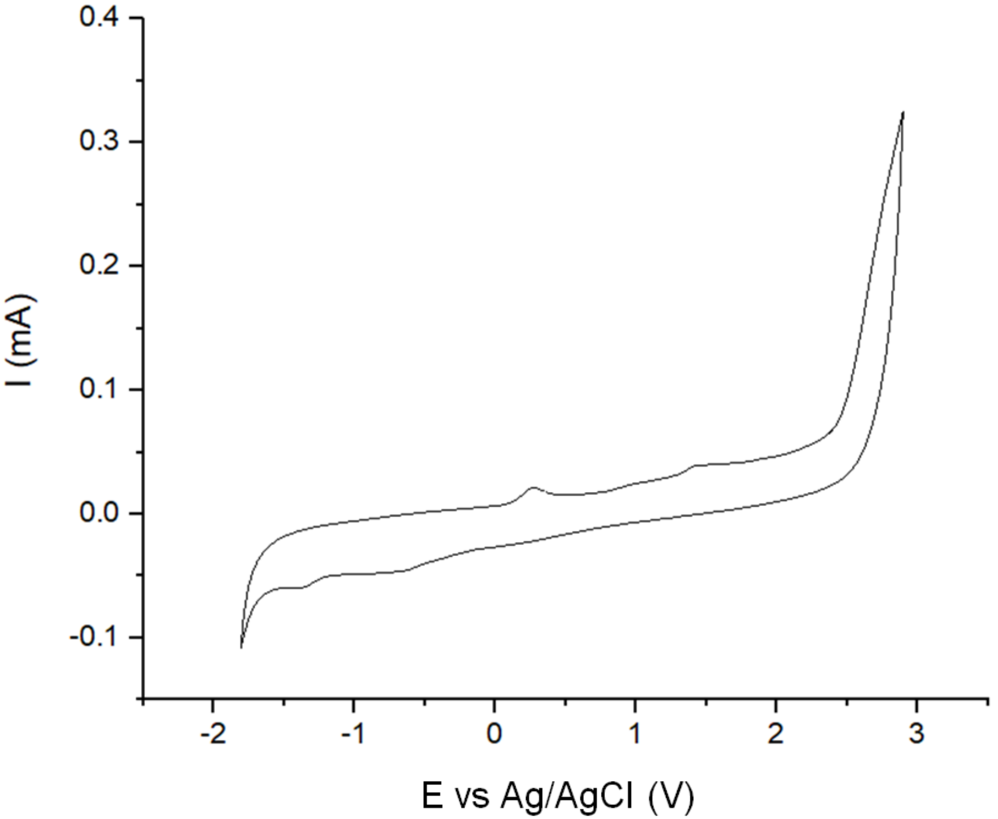

Figure S41. Cyclic voltammetry of compound **7**.

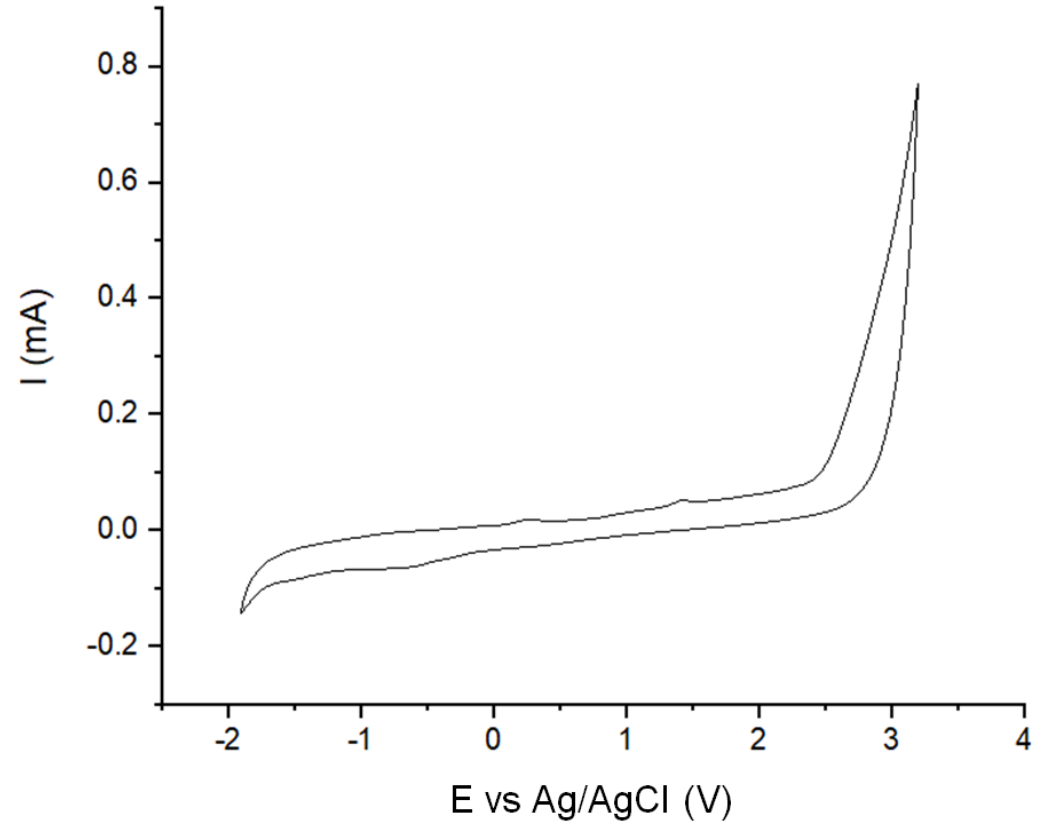

Figure S42. Cyclic voltammetry of compound **8**.
